# Effects of Immersive Virtual Reality on Upper-Extremity Stroke Rehabilitation: A Systematic Review with Meta-Analysis

**DOI:** 10.3390/jcm13010146

**Published:** 2023-12-27

**Authors:** Pawel Kiper, Nathalie Godart, Manon Cavalier, Charlotte Berard, Błażej Cieślik, Sara Federico, Aleksandra Kiper, Leonardo Pellicciari, Roberto Meroni

**Affiliations:** 1 Healthcare Innovation Technology Lab, IRCCS San Camillo Hospital, 30126 Venice, Italysara.federico@hsancamillo.it (S.F.); 2 Department of Physiotherapy, LUNEX International University of Health Exercise and Sports, L-4671 Differdange, Luxembourg; godart.nathalie@stud.lunex-university.net (N.G.);; 3 Doctoral School of the University of Rzeszów, University of Rzeszów, 35-959 Rzeszów, Poland; 4 IRCCS Istituto delle Scienze Neurologiche di Bologna, 40139 Bologna, Italy; leonardo.pellicciari@ausl.bologna.it; 5 Luxembourg Health & Sport Sciences Research Institute ASBL, L-4671 Differdange, Luxembourg

**Keywords:** immersive VR, stroke, upper extremity, virtual reality, hand functions

## Abstract

Virtual reality (VR) is an innovative rehabilitation tool increasingly used in stroke rehabilitation. Fully immersive VR is a type of VR that closely simulates real-life scenarios, providing a high level of immersion, and has shown promising results in improving rehabilitation functions. This study aimed to assess the effect of immersive VR-based therapy for stroke patients on the upper extremities, activities of daily living (ADLs), and pain reduction and its acceptability and side effects. For this review, we gathered all suitable randomized controlled trials from PubMed, EMBASE, Cochrane Library, Scopus, and Web of Science. Out of 1532, 10 articles were included, with 324 participants. The results show that immersive VR offers greater benefits in comparison with conventional rehabilitation, with significant improvements observed in ADLs (SMD 0.58, 95% CI 0.25 to 0.91, I^2^ = 0%, *p* = 0.0005), overall function as measured by the Fugl-Meyer Assessment (MD 6.33, 95% CI 4.15 to 8.50, I^2^ = 25%, *p* = 0.00001), and subscales for the shoulder (MD 4.96, 95% CI—1.90–8.03, I^2^ = 25%, *p* = 0.002), wrist (MD 2.41, 95% CI—0.56–4.26, I^2^ = 0%, *p* = 0.01), and hand (MD 2.60, 95% CI—0.70–4.5°, I^2^ = 0%, *p* = 0.007). These findings highlight the potential of immersive VR as a valuable therapeutic option for stroke survivors, enhancing their ADL performance and upper-limb function. The immersive nature of VR provides an engaging and immersive environment for rehabilitation.

## 1. Introduction

A stroke is a sudden interruption of the blood supply to the brain, resulting in brain damage, causing disability and mortality [1]. It is one of the leading causes of acquired adult disability and the second to the third most common cause of mortality due to its high prevalence [2]. Recovery is thought to be possible within the first few weeks after the initial stroke, and long-term functional improvements can occur even months after the stroke [2]. A reduced capacity to conduct activities of daily living (ADLs) and sensory, cognitive, and motor impairments, as well as a decline in social and community participation, constitute a significant component of stroke-related disability [3]. The best strategy for restoring upper-limb function is meaningful, consistently challenging, and highly repetitive training [4,5].

Virtual reality (VR) is a hardware- and software-based computer–human interface system providing a multimodal, stimulating environment through three-dimensional support and offering realistic ADL scenarios [6]. It enhances feedback by stimulating the visual, vestibular, and somatosensory systems through engagement with a digital reality environment [6]. Immersion and presence are the two main concepts of VR, and we can distinguish four different degrees of virtual immersion: mixed VR, augmented VR, immersive VR, and non-immersive VR. Participants’ subjective perception of presence in the virtual world depends on their level of immersion, which is connected to the virtual system [7,8]. Mixed VR incorporates elements of both the virtual and physical worlds, seamlessly blending digital and real-world stimuli [9]. Augmented VR overlays digital content onto the user’s view of the physical environment, enhancing real-world experiences with additional information [9]. Non-immersive VR, on the other hand, such as videos or body representations on traditional screens, lacks the depth of full immersion, providing a more basic digital experience without extensive sensory engagement [10].

Immersive VR, the focus of this study, is a newer type of VR that places the participant in a three-dimensional world. It includes a head-mounted display with visual and aural signals and controllers that use haptic (sense of touch) feedback [11]. Compared to older VR technologies, immersive VR offers a more lifelike environment scene design and precise object tracking. It also provides real-time feedback through vision, touch, and hearing and permits motion in real-life ADL scenarios to rebuild physical function [12].

Immersive VR therapy has shown multiple benefits in restoring upper- and lower-limb motor function in stroke patients [13]. It allows patients to complete more repetitive functional tasks than conventional therapy [14] and can be utilized more as a game than a treatment, increasing treatment adherence and the motivation to adhere to the treatment [13]. Moreover, VR facilitates the acquisition of enhanced feedback, a crucial motor-learning mechanism for neurological patients [15]. By offering real-time information on performance and aiding individuals in refining and adjusting their movements, VR contributes to a dynamic learning process, providing more nuanced insights into their performance and ultimately enhancing the effectiveness of rehabilitation strategies [15].

The application of VR in neurological rehabilitation settings is expanding rapidly [16]. The existing reviews primarily focus on meta-analyses concerning the utilization of non-immersive VR or systematic reviews without meta-analysis, contributing to a nuanced understanding of VR’s efficacy for diverse patient populations [17,18]. While existing studies have explored the impact of VR systems on the upper limbs of stroke patients, the lack of differentiation between types of immersion reveals a gap in our knowledge, highlighting the need for further investigation into how these immersion levels influence treatment effectiveness [19]. Moreover, the potential side effects of VR systems, such as dizziness and eyestrain, associated with immersive VR experiences have yet to be comprehensively assessed [20]. Understanding these potential side effects is crucial for ensuring the safety and well-being of individuals undergoing therapeutic interventions. Despite promising results, the absence of a distinction between VR systems and the unknown side effects of this treatment emphasize the necessity for further research to refine our understanding of immersive VR’s role in therapeutic interventions.

This review’s primary aim was to assess immersive VR’s effectiveness on upper-limb function, ADLs, and pain reduction. The secondary purpose was to appraise immersive VR’s side effects and acceptability. The following review questions were posed: Does immersive VR improve upper-extremity function, strength, and dexterity in people after a stroke? Does immersive VR improve ADLs and reduce pain? Is immersive VR an acceptable form of post-stroke rehabilitation?

## 2. Materials and Methods

### 2.1. Design and Protocol Registration

This study was designed as a systematic review with meta-analysis. The protocol of this review was registered a priori with the PROSPERO database (CRD42023393266). The Preferred Reporting Items for Systematic Reviews and Meta-Analyses (PRISMA) Statement was followed for reporting [21].

### 2.2. Search Strategy and Study Selection

The collection of published articles followed a three-stage process. In the first stage, a preliminary search was conducted in PubMed to identify appropriate keywords in titles and abstracts. In the second stage, the search was expanded using the specified keywords and their synonyms, and adjustments were made as necessary to suit each selected database. Articles found using the search in the second stage were collected through their reference lists. All articles were included without date restrictions if they were available in English. The databases used to collect articles were PubMed, EMBASE, Cochrane Library, Scopus, and Web of Science (Supplementary Materials S1). The search was last conducted on 15 November 2023.

For data synthesis, the titles and abstracts of eligible articles were collected using Rayyan software (Qatar Computing Research Institute, Qatar; www.rayyan.ai (accessed on 1 January 2023)). Two reviewers independently screened studies and selected those that met the inclusion criteria. Any disagreements in abstract screening were solved through discussion with a third reviewer. Subsequently, full texts were obtained for the included abstracts. Two reviewers evaluated the quality of the full texts, and a third reviewer resolved any disagreements.

### 2.3. Eligibility Criteria

This study only included randomized controlled trials that involved participants who suffered from a stroke, regardless of the stage of stroke (i.e., acute, subacute, or chronic). The intervention groups in the selected trials used immersive VR-based rehabilitation, while robotic interventions and exoskeletons were excluded from the intervention of interest. The control groups (CGs) received conventional physiotherapy. The primary outcome of interest assessed was upper-extremity function. The secondary outcomes of interest covered hand dexterity, hand strength, grip strength, ADLs, upper-extremity function and performance, coordination, functional mobility, pain, and adverse effects.

### 2.4. Risk-of-Bias Assessment

Two authors independently assessed the methodological quality of the included trials using the Cochrane risk-of-bias tool (RoB 2.0) [22]. Disagreements were resolved by involving a third author. For RoB 2.0, the domains (i.e., randomization process, deviations from the intended intervention, missing outcome data, measurement of the outcome, and results reporting) were assessed to obtain, for each study, an overall risk-of-bias judgment that ranged from low (i.e., when all domains have a low risk of bias) to high (i.e., when the study has at least one domain with a high risk of bias or multiple domains showing biasing concerns). We contacted the authors of the studies included in cases of missing data. Finally, the presence of potential publication bias was investigated through a visual inspection of funnel plots.

### 2.5. Results Analysis

A narrative synthesis of the findings from the studies included was performed. A potential meta-analysis of quantitative data was run with available data. A narrative review was conducted to analyze statistical differences and identify significant results among the included outcome measures, using a significance level of *p*-value  <  0.05. A quantitative meta-analysis was also performed using RevMan software version 5.4, incorporating the mean, standard deviation, and sample size. The mean difference (MD)—in the case of the same outcome among the meta-analyzed studies—and standardized mean difference (SMD)—in the case of different outcomes among the meta-analyzed studies—with a 95% confidence interval (CI) were calculated to assess the overall results. Forest plot graphics were generated to demonstrate the pooled effect. Heterogeneity was assessed using the I^2^ statistic and was categorized as low if I^2^ < 25%, moderate if I^2^ was between 25 and 50%, and high if I^2^ > 50% [23].

### 2.6. Grade and Quality of Evidence

The evaluation of evidence quality is implemented through the GRADE assessment [24]. This method entails individually grading the certainty of evidence for each outcome deemed significant to patients, followed by determining an overarching certainty across all outcomes. This reflects our confidence in the accuracy of the effect estimate. Additionally, the GRADE approach advocates for the selection of all patient-relevant outcomes and assigns importance ratings to each. To facilitate outcome ranking based on importance (refer to “Importance” in Table 1), numerical ratings on a 9-point scale were used (7 to 9—critical; 4 to 6—important; 1 to 3—of limited importance). Table 1 provides outcome-specific details on overall evidence quality. We assessed the evidence for study limitations (risk of bias), result inconsistency, evidence indirectness, effect estimate imprecision, and potential publication bias (other considerations). The functional upper-limb outcome was evaluated using the FMA-UE scale. For transparency, the GRADE system categorizes evidence certainty into four grades: (1) high—further research is unlikely to significantly alter our confidence in the effect estimate; (2) moderate—further research is likely to have a substantial impact on our confidence and may alter the estimate; (3) low—further research is highly likely to significantly impact our confidence and is likely to change the estimate; (4) very low—any effect estimate is highly uncertain.

## 3. Results

### 3.1. Study Selection

**Figure 1:**
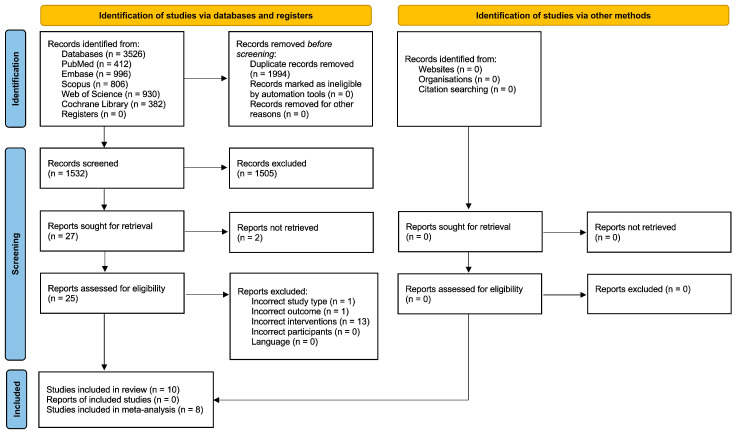
PRISMA Flow Diagram illustrating the systematic review procedures. The diagram depicts the flow of information from the initial identification of records through database searching to the final inclusion of studies in the qualitative and quantitative synthesis.

The search was conducted across multiple databases, yielding 3526 search results. Duplicate articles were subsequently removed, resulting in a total of 1532 articles. The articles were allocated to two reviewers to screen the titles and abstracts. Based on predetermined eligibility criteria, 1505 articles were excluded, leaving 27 for full-text review. Two of the studies were not retrieved, leaving a total of 25 articles to be assessed by the reviewers. Thirteen studies were excluded as the VR system was not fully immersive, one was excluded due to incorrect outcomes, and one was excluded because it was not a randomized controlled trial (Supplementary Materials S2). Finally, ten studies were included in the systematic review, and eight were included in the meta-analysis [12,25,26,27,28,29,30,31]. A PRISMA flow diagram was created to represent this process, as shown in Figure 1.

### 3.2. Study Characteristics and Results Summary

Table 2 provides a comprehensive depiction of the characteristics of the included studies. In the study by Chatterjee et al. [25], the authors developed a program named VIRTUE. The use of VIRTUE demonstrated favorable safety outcomes, and the program’s acceptability was higher in the VR group. The authors associated positive reductions in hospital stays for all patients with VR treatment. The sham group showed significant differences from the severe- and moderate-cognitive-impairment groups, with *p*-values of 0.03 and 0.03, respectively. In both groups, patients in the subacute post-stroke stage were included. The only significant difference in the secondary outcome was decreased anxiety levels in the group with mild to moderate cognitive impairment.

In Choi et al.’s [26] study, patients in the subacute post-stroke stage were assigned to the digital practice group and received VR-based training in a Leap Motion environment. The VR group improved in the line bisection test (*p* = 0.02). Relative to the control group, the VR group exhibited significantly higher values concerning the mean change in Motor-Free Visual Perception Test Vertical Version raw scores (*p* = 0.02), reaction behavior left (*p* = 0.02), performance behavior left (*p* = 0.02), performance behavior right (*p* = 0.014), and processing time (*p* = 0.01). The VR group showed significantly more progress than the control group in the head rotation degree and velocity (*p* = 0.07 and *p* = 0.001, respectively).

In the study by Hsu et al. [27], participants in the chronic stage of stroke were randomly assigned to one of three groups: conventional occupational therapy, mirror therapy, and VR-based therapy. The wrist sub-score of the FMA-UE (*p* = 0.01) and the Box and Block Test (BBT) (*p* = 0.04) showed statistically significant group-by-time interaction effects. VR-based mirror therapy appeared to have promising benefits for regaining upper-extremity motor function in chronic stroke patients.

In Huang et al.’s [28] study, chronic stroke patients were allocated to either VR-based motor control training or the standard occupational therapy group. Time significantly affected every clinical outcome (*p* = 0.05) except for FMA-UE-Coordination/Speed (*p* = 0.05). Significant differences were observed in AROM-Elbow Extension (*p* = 0.007), and active range of motion (AROM-Forearm Pronation) (*p* = 0.048) items showed significant effects within groups. AROM-Shoulder Flexion (*p* = 0.001), FMA-UE-Shoulder/Elbow/Forearm (*p* = 0.004), and FMA-UE-Total score (*p* = 0.008) all had significant associations between time and group. Side effects were reported using the Simulator Sickness Questionnaire (including 18 possible side effects of immersive VR). They found that some participants had moderate eye discomfort (strain and blurred vision) and sweating after training. Still, participants tended to be highly satisfied with VR training (average of 4.5 out of 5 points).

In the study by Lin et al. [29], chronic stroke patients with mild to severe hemiparesis were enrolled in two groups: the mirror therapy group and the VR-mirror therapy group (VRMT). Significant effects were seen on the hand subsection of the FMA (*p* = 0.008) and the total score of the FMA (*p* = 0.03) in the VRMT group.

In the study by Mekbib et al. [30], participants were assigned to occupational therapy alone or with VR groups. Both groups included individuals in the subacute stroke stage and demonstrated a significant increase in the BI (*p* < 0.05). The VR group exhibited a noteworthy improvement in FMA-UE scores (*p* < 0.05).

Ögün et al. [12] investigated the effect of an immersive VR device on upper-extremity function in patients with ischemic chronic stroke. Participants were randomly assigned to either the VR or control group. The results revealed that the VR group had significantly higher scores than the control group in FMA-UE, the Action Research Arm Test (ARAT), the Functional Independence Measure (FIM), and the Performance Assessment of Self-Care Skills (PASS) (*p* < 0.001).

In Song et al.’s [31] study, chronic stroke participants were randomly allocated to the VR-based bilateral arm training group or the normal bilateral arm training group. Both groups significantly improved their scores on the manual function test. The virtual and control groups had *p*-values of 0.042 and 0.039, respectively. The only difference between the two groups was observed when using the proprioception test (*p* = 0.04).

In Huang et al.’s [32] study, participants in the experimental group received 30 min of conventional rehabilitation followed by 30 min of immersive VR sessions for three weeks. The control group received 60 min of daily conventional rehabilitation. The authors concluded that immersive-VR-based rehabilitation is an effective tool that can improve the recovery of the UE functional capabilities (FMA-UE) of subacute stroke patients when added to standard care.

Sip et al. [33] assessed immersive VR as a physical therapy option for restoring post-stroke hand function. The study compared an experimental group (n = 10) receiving VR therapy using the SciMed system and Virtual Mirror Hand 1.0 and a control group (n = 10) undergoing classic mirror therapy. Both groups had 18 sessions, six days per week, for three consecutive weeks. No significant differences were found between treatments in FMA-UE, but VR therapy participants reported improved pain and subjective sensations.

No articles included in the study examined or evaluated pain as an outcome measure. One article [29] reported adverse events, defined as a headache, dizziness, nausea, or blurred vision. The authors stated that the reported adverse effects could be attributed to the intervention and required a visit to a hospital.

### 3.3. Meta-Analysis Results

We conducted a meta-analysis to compare the effects of immersive VR with conventional therapy by investigating the effectiveness of the intervention in improving various assessment tools related to upper-extremity functions. These tools included subscales focusing on the upper extremities, ADLs, and the upper-extremity section of the Fugl-Meyer Assessment. Analyses were performed with the mean difference (MD) and a fixed-effects model. To gather additional data for further analysis, we attempted to reach out to the authors of the relevant studies. However, we did not receive any responses from them.

#### 3.3.1. Function Assessed by the Fugl-Meyer Assessment for Upper Extremity

**Figure 2:**
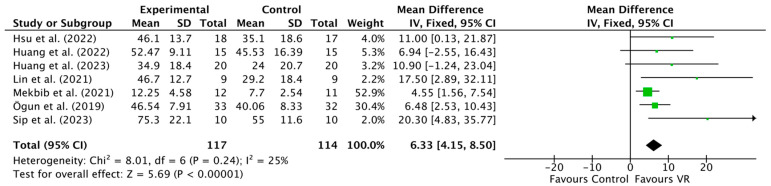
Comparison: virtual reality (VR) versus control group (CG); outcome: upper-extremity function post-intervention analysis [12,27,28,29,30,32,33].

Seven studies [12,27,28,29,30,32,33] analyzed the effect of VR on upper-extremity function using the Fugl-Meyer Assessment. The findings of this meta-analysis (Figure 2) demonstrate a significant positive effect of VR interventions on upper-extremity function measured by the Fugl-Meyer Assessment compared to the control group (MD 6.33, 95% CI 4.15 to 8.50, I^2^ = 25%, *p* = 0.00001, 231 participants overall).

#### 3.3.2. Upper-Extremity Analysis

**Figure 3:**
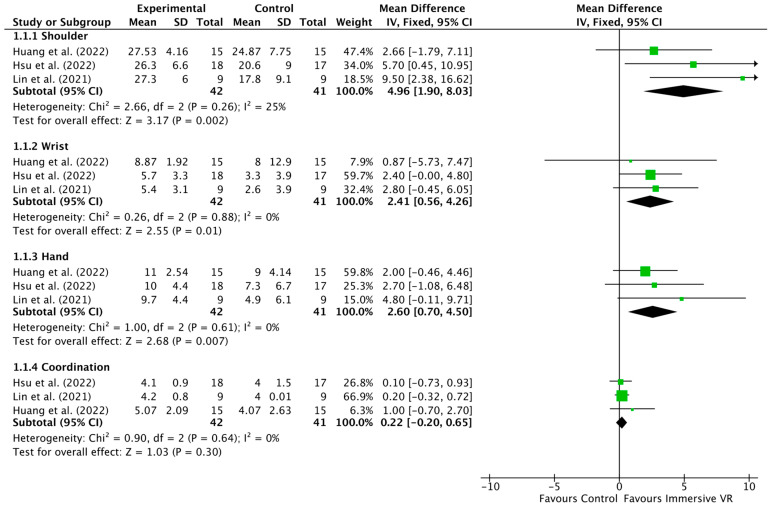
Comparison: VR versus CG; outcome: Fugl-Meyer upper-extremity subscale post-intervention analysis [27,28,29].

The results indicate that the VR groups significantly improved compared to the control group in the shoulder, wrist, and hand FMA subscales. Figure 3 displays the results of the meta-analyses, with a breakdown of subscales for the shoulder, wrist, hand, and coordination of movement.

Three studies examined the effect of VR on shoulder function by using the Fugl-Meyer Assessment subscale [27,28,29]. The results show that shoulder function improved significantly in the VR group compared to the control group (MD 4.96, 95% CI—1.90 to 8.03, I^2^ = 25%, *p* = 0.002, 83 participants). Similarly, for the Fugl-Meyer Assessment wrist subscale, there is a statistically significant improvement in wrist outcomes for the VR group compared to the control group (MD 2.41, 95% CI—0.56 to 4.26, I^2^ = 0%, *p* = 0.01, 83 participants). Regarding the hand subscale, there is a statistically significant improvement in hand outcomes for the VR group compared to the control group (MD 2.60, 95% CI—0.70 to 4.5°, I^2^ = 0%, *p* = 0.007, 83 participants). Finally, the meta-analysis for coordination did not show a significant difference between the VR and control groups (MD 0.22, 95% CI –0.20 to 0.65, I^2^ = 0%, *p* = 0.30, 83 participants).

The findings of this review demonstrate that VR interventions had a positive impact on shoulder, wrist, and hand outcomes compared to the control group, while coordination outcomes showed comparable effects. The heterogeneity among studies was low to moderate, indicating some variability in the results of the included studies for the shoulder Fugl-Meyer Assessment subscale analysis.

#### 3.3.3. Activities-of-Daily-Living Analysis

**Figure 4:**
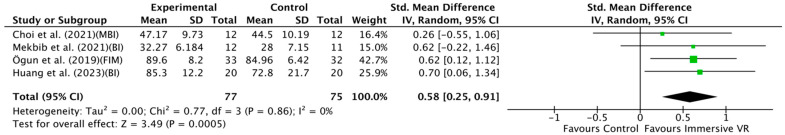
Comparison: VR versus CG; outcome: activities-of-daily-living post-intervention analysis [12,26,30,32].

The results shown in Figure 4 from the four studies [12,26,30,32] that analyzed the impact of VR therapy on ADLs highlight that VR interventions had a significant positive effect on ADL outcomes compared to the control group (SMD 0.58, 95% CI 0.25 to 0.91, I^2^ = 0%, *p* = 0.0005, 152 participants).

### 3.4. Risk-of-Bias Assessment

**Figure 5:**
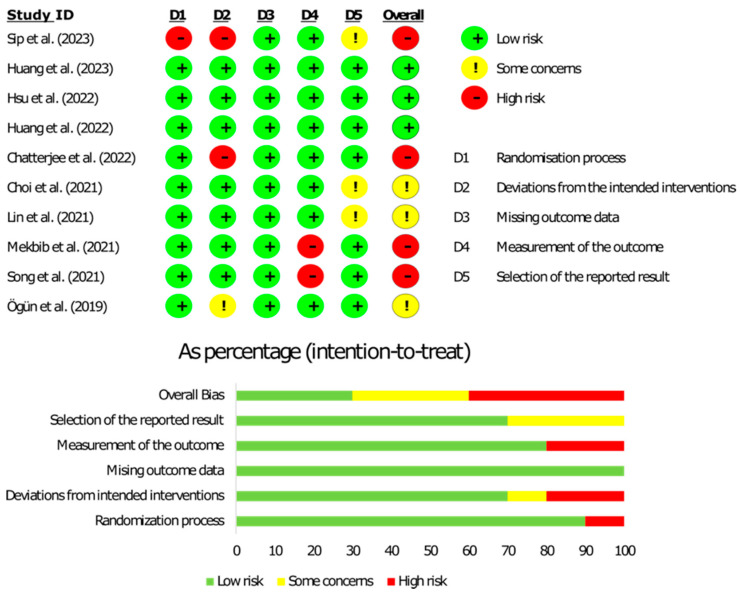
Risk of bias in included studies [12,25,26,27,28,29,30,31,32,33].

For each of the ten randomized controlled trials included in this systematic review, the risk-of-bias assessment is shown in Figure 5 and Table S1 (Supplementary Materials S3). The randomization process and missing outcome data were considered to have a low bias risk for nine studies and a high risk for one study. Participants were all randomly allocated using a sequence generation process, with independent enrollment personnel controlling the allocation process. Studies did not show missing data for all participants randomly allocated. The selection of the reported results was examined, and the risk of bias was found in three papers [26,29,33] to be of some concern due to a lack of trial registration. Two studies had a high risk of bias in measuring the outcome [30,31]. The assessors could have impacted the outcome, and the outcome measurement could have differed between groups. Deviations from the intended intervention were reported as having a high risk of bias for two out of ten studies [25,33] and being of some concern for one out of ten studies [11] due to deviations from the intended intervention that could have affected the outcome. Overall, three out of ten studies were marked as low risk [27,28,32], three out of ten studies were considered to have some concerns of bias [12,26,29], and four out of ten studies were determined to have a high risk of bias [25,30,31,33]. Figure S1 (Supplementary Materials S3) illustrates the funnel plot of the included studies.

## 4. Discussion

This review explored the impact of immersive VR versus conventional therapy on upper-extremity function, ADLs, and pain reduction and the side effects and acceptability of immersive VR. Due to a lack of data, we analyzed the impact of immersive VR on upper-limb function and ADLs among the primary and secondary objectives of the review. The inclusion criteria did not specify a publication year, but all selected papers were published within the last four years, indicating the recent emergence of immersive-VR-assisted therapy in the field. Fully immersive VR treatment has significant promise in functional stroke recovery and may provide additional benefits over traditional therapy on upper limbs and ADLs [13,17].

The interpretation of the findings of this systematic review on immersive VR interventions in stroke patients should be contextualized considering the varying degrees of bias in the individual studies, of which only three out of ten were classified as low risk. Thus, the variability in the risk of bias across studies necessitates exploration. Standardization strategies for future research could involve refining inclusion criteria, ensuring consistent methodologies, and establishing clear reporting guidelines. By addressing these variations, the reliability and comparability of studies can be improved, facilitating a more comprehensive understanding of immersive VR’s effectiveness in stroke rehabilitation. The results of the included studies were mixed, with some studies reporting positive effects of VR interventions on various outcome measures, such as safety [25,28], acceptability [25], reductions in hospital stays [25], improvements in cognitive impairment [26], the line bisection test [26], visual perception tasks [26], wrist motor function [27,28,29,31], upper-extremity motor function [12,28], side effect [28] ADLs [12,25,30], and Fugl-Meyer assessment scale scores [12,27,28,29,30].

The meta-analysis of eight studies examining the effect of VR interventions on upper-extremity function using the Fugl-Meyer Assessment test showed a significant positive effect. Looking at the Fugl-Meyer Assessment subscales, VR interventions positively impacted shoulder, wrist, and hand outcomes compared to the control group, while coordination outcomes showed comparable effects. The heterogeneity among studies was low to moderate. Additionally, four studies analyzing the impact of VR therapy on ADLs showed that VR interventions had a significant positive impact on ADL outcomes compared to the control group. Improved motor function and ADL performance hold significant clinical relevance, potentially reshaping rehabilitation protocols. Clinicians may consider incorporating immersive VR interventions that are particularly tailored to address specific deficits in shoulder, wrist, and hand function. These findings offer a basis for refining rehabilitation strategies, emphasizing targeted exercises for enhanced outcomes in upper-extremity recovery. The lack of significant improvement in coordination outcomes between the VR intervention groups and the control groups could be attributed to the level of shoulder, wrist, and hand function not reaching the threshold necessary to observe substantial changes in coordination abilities. To obtain substantial improvements in coordination, it may be necessary to design interventions that specifically target coordination skills or provide interventions that address a broader range of upper-extremity functions to reach a higher functional recovery overall.

Further research and intervention refinement are needed to better understand and enhance coordination outcomes in stroke patients. From a broader perspective, these findings contribute to evidence indicating the beneficial effects of VR interventions on upper-extremity functions. The results support immersive VR’s effectiveness in improving motor function and ADL performance. Nonetheless, it is crucial to acknowledge the potential influence of biases in individual studies, as these factors may impact the generalizability of the findings.

Arm–hand motor dysfunction is typical after a stroke; over half of those with hand deficits never regain function [34]. The hands play a crucial role in our daily lives, providing us with dexterity and coordination to perform essential tasks [35]. Using our hands effectively is fundamental for object handling, as it involves stabilization and manipulation. Object manipulation requires the coordinated movement and control of our fingers, hands, and wrists to grasp, hold, and manipulate objects of various shapes and sizes [36]. This level of dexterity allows us to perform intricate tasks such as writing, typing, cooking, and playing musical instruments.

Furthermore, the bilateral use of our upper limbs is a common occurrence in many activities. From simple actions like carrying groceries or opening a jar to more complex tasks such as driving or playing sports, the coordinated use of both hands is often required for optimal performance and efficiency [37]. In contrast, if our lower limbs are impaired or non-functional, we can often rely on adaptive devices like wheelchairs or prosthetics to regain mobility and independence. However, the same level of adaptability is not readily available for individuals with functional impairments in their upper limbs. The loss of upper-limb function presents significant challenges, as it limits our ability to perform daily tasks that require precise hand movements and coordination [35]. To compensate for the loss of functional upper limbs, individuals may need to rely on assistive tools and devices, which can vary depending on the specific tasks and individual needs [38]. However, these adaptations may not fully restore the natural functionality and versatility of the hands [37]. Given the crucial role of hand dexterity and coordination in daily activities, interventions like VR therapy that aim to improve upper-limb function hold significant value in enhancing the quality of life for individuals with upper-limb impairments [39]. By improving shoulder, wrist, and hand outcomes, interventions aim to restore functional capabilities and promote independence in performing essential tasks that rely on dexterity and coordination [38].

The novelty of immersive VR in stroke rehabilitation represents a pivotal aspect influencing our findings. The continuous evolution of technology in healthcare introduces innovative approaches, and immersive VR stands out as a promising tool. Its impact is evident in the enhanced upper-extremity function and improved ADL outcomes observed in our study. The correlation with technological evolution suggests that integrating immersive VR into rehabilitation protocols can offer novel and effective interventions for stroke recovery.

The study by Hao et al. (2023) contributes valuable insights into the effectiveness of immersive VR in post-stroke rehabilitation, particularly focusing on upper-extremity function [40]. Their results, based on a network meta-analysis of twenty randomized controlled trials with 813 participants, indicate that immersive VR systems were the most effective in improving upper-extremity function, surpassing non-immersive VR systems and non-immersive gaming consoles. Conventional rehabilitation, in comparison, was identified as the least effective approach. Considering our results on VR interventions and stroke rehabilitation, these findings align with the growing, but still limited, body of evidence supporting the efficacy of immersive VR in promoting upper limb recovery post-stroke. This aligns well with the emerging consensus in the field and reinforces the significance of our findings in contributing to the existing body of knowledge. It is noteworthy that both our review and the findings by Hao et al. collectively contribute to the understanding of the therapeutic potential of immersive VR in post-stroke rehabilitation, further strengthening the evidence base for the adoption of these interventions in clinical practice.

Additionally, the results of Jin et al.’s (2022) study support the use of VR for improving motor impairment and activities of daily living after a stroke [41]. The recommendation is particularly favorable for patients with moderate-to-severe arm paresis, suggesting that they can make more progress through VR training. The emphasis on the immersive design of VR interventions is noteworthy, with immersive virtual reality shown to produce a greater beneficial effect compared to other forms of VR. The acknowledgment of the superiority of immersive VR experiences for patients with moderate-to-severe arm paresis aligns well with the evolving understanding of tailored interventions for specific subgroups within the stroke population. These findings not only underscore the clinical relevance of incorporating VR into rehabilitation protocols but also emphasize the importance of considering the immersive nature of VR interventions for optimal outcomes, especially for patients with moderate-to-severe arm paresis.

Based on our findings, clinicians can be encouraged to consider the integration of VR interventions into stroke rehabilitation protocols to enhance overall upper-limb functionality. Specific improvements in shoulder, wrist, and hand function are highlighted within the VR groups compared to controls, as well. This information serves as a guide for clinicians to tailor interventions, emphasizing targeted exercises to address deficits in these specific areas for more effective outcomes. The analysis of VR therapy’s impact on ADLs indicates a significant positive effect. Clinicians may find value in incorporating VR interventions not only to improve upper-extremity function but also to enhance the broader aspect of functional independence in daily life. The collective findings suggest that VR interventions contribute to a comprehensive improvement in upper-extremity function and ADL outcomes. Therefore, clinicians are encouraged to explore VR as a valuable tool in holistic stroke rehabilitation, addressing both specific motor deficits and the broader spectrum of functional abilities.

Future research directions could address the limitations identified in the previous studies, such as by improving the study designs, reducing bias risks, addressing heterogeneity, and conducting long-term follow-up studies. Further investigations could explore the specific mechanisms through which VR interventions influence upper-extremity functions and identify the optimal protocols, dosage, timing, and adverse effects of immersive VR interventions for different subgroups of stroke patients. Investigating the adverse effects of fully immersive VR interventions on stroke patients is important to ensure patient safety and understand potential risks associated with using this technology in rehabilitation. Future studies should also consider incorporating pain assessment as an important outcome measure when studying the effects of immersive VR. Understanding the potential impact of VR on pain management can provide valuable insights for improving therapeutic interventions and the overall patient experience. By exploring the relationship between VR and pain outcomes, researchers can contribute to developing more comprehensive and effective treatment approaches in healthcare settings. Moreover, there is a need for studies focusing on patient preferences and adherence to VR-based interventions, considering the subjective experiences and perspectives of individuals undergoing such rehabilitation. Further investigations may also assess the transferability of skills acquired through VR training into real-world functional activities, providing a more comprehensive understanding of the long-term impact of immersive VR interventions on daily life and functional independence.

Additionally, investigating the effects of VR on pain can help identify its potential as a non-pharmacological intervention for pain management, offering alternative options for individuals suffering from various pain conditions. Conducting studies that include comprehensive long-term follow-up assessments would provide valuable insights into the sustained benefits and functional outcomes of VR training. This would contribute to evidence-based decision making in clinical practice and inform the development of targeted rehabilitation interventions for stroke survivors.

Our review possesses several strengths that enhance its significance and reliability in exploring immersive VR interventions. Firstly, our study adopts a focused approach by specifically examining the impact of immersive VR on upper-extremity function, delving into shoulder, arm, and hand function. This focused strategy allows for a more in-depth and detailed analysis within a specific domain of stroke rehabilitation, providing valuable insights into the effectiveness of immersive VR interventions. Secondly, our findings hold practical relevance, as they underscore the potential of immersive VR in promoting upper-extremity recovery after stroke. This clinical significance offers actionable insights that can guide clinicians in optimizing interventions for improved patient outcomes. Lastly, while we acknowledge the limitation of having limited data to analyze other aspects related to our objectives, such as pain and adverse events, this indicates a gap in the literature. By highlighting this gap, we invite the research community to explore these important aspects, which can significantly influence stroke recovery. This review is subject to some limitations that should be acknowledged. Firstly, the presence of bias in the studies included is a notable concern. Among the ten included randomized clinical trials, only three studies were determined to have a low risk of bias. Thus, these varying degrees of bias must be considered when interpreting the findings. Secondly, the limited number of papers included in this study may also restrict the generalizability of the results and the ability to draw definitive conclusions. A larger pool of studies would provide a more comprehensive understanding of the effectiveness of immersive VR interventions for upper-extremity functions in stroke patients. Although efforts were made to obtain additional data from the authors of the studies, the limited data availability for analysis is another constraint. Thirdly, the number of participants in the studies was generally low. The findings may not be representative of the larger population. The limited number of participants reduces the statistical power and generalizability of the results. Finally, another limitation is the absence of long-term follow-up data in the studies included. The sustainability of the improvements observed with immersive VR interventions and the transfer of skills to real-world functional activities remain uncertain.

## 5. Conclusions

The current review’s findings support the use of immersive VR in stroke patients’ upper-limb rehabilitation plans. The meta-analysis demonstrated that immersive VR interventions significantly positively impacted shoulder, wrist, and hand outcomes compared to control groups. However, no significant improvement was observed in coordination outcomes. Additionally, significant improvements in ADL outcomes were found for the VR group. None of the selected studies provided data on whether immersive VR reduced post-stroke pain, and only one study noted moderate side effects using immersive VR.

## Figures and Tables

**Table 1:** Immersive VR compared to conventional physiotherapy for upper-limb function in people after stroke.

Certainty Assessment							n of Patients		Effect		Certainty	Importance
n of Studies	Design	Risk of Bias	Inconsistency	Indirectness	Imprecision	Other Considerations	iVR	CR	Relative (95% CI)	Absolute (95% CI)		
Upper-limb function improvement (assessed with FMA-UE; scale from 0 to 66)												
7	RCTs	Serious ^a^	Not serious	Not serious	Serious ^b^	None	117	114	-	MD 6.33 effect higher (4.15 higher to 8.5 higher)	⨁⨁◯◯ Low	Important

iVR: immersive virtual reality; CR: conventional rehabilitation; RCTs: randomized controlled trials; CI: confidence interval; MD: mean difference; FMA-UE: Fugl-Meyer Assessment for upper extremity; ^a^ concerns related to measurement of the outcomes and to the reporting of the results; ^b^ small number of studies and wide CI.

**Table 2:** Detailed characteristics of the included studies.

Study ID	Author (Year)	Study Design	Aim	Group Characteristics	Interventions	Outcome Measures	Timepoints	Conclusions
1	Huang et al. (2023) [32]	RCT	To assess the extent to which imVR-based UE rehabilitation could enhance conventional treatment. Additionally, the study aimed to investigate changes in brain functional connectivity associated with the rehabilitation process.	Subjects aged 30 to 85 years old, first stroke event within 1 month prior to enrollment, subacute stage with a subcortical lesion (basal ganglia, internal capsule, corona radiata, or brainstem), starting UL function of Brunnstrom stages II–IV. Exclusion criteria: History of transient ischemic attack, failure of critical organs, previous brain neurosurgery or epilepsy, severe cognitive impairments, or aphasia; subjects not suitable for an MRI scan or enrolled in another clinical trial involving PT or an investigational drug. EG = imVR, age 63.3 (14.3), 13 M, 7 F; CG = conventional rehabilitation, age 65.1 (6.1), 11 M, 9 F.	Both groups received rehabilitation training 5 days/wk for 3 wks. EG = 30′ of conventional rehabilitation + 30′ imVR system rehabilitation (environments: frying dumplings and noodles, popping balloons with a virtual sword, punching dolls in a virtual boxing arena, playing basketball in a virtual court, collecting eggs into a virtual basket, and tidying up a desk in a virtual office). The VR system comprised HMD—HTC Vive-VR; wireless controllers; 2 base stations using steamVR^®^ technology; and computer. CG = 60′ conventional rehabilitation program daily. Design aimed for similar intensity and complexity to the immersive VR group. Program included PT and OT, covering grips, selective finger movements, gross movement, strength training, stretching, and ADLs.	FMA-UE, BI, RS-fMRI, FC, ADL, MRI, TR/TE, FOV, mFD, FLIRT, FNIRT, MNI152	T0: baseline T1: post-intervention (wk 3) T2: follow-up (12 wks after T1)	ImVR-based rehabilitation is an effective tool that can improve the recovery of UE functional capabilities of subacute stroke patients when added to standard care.
2	Sip et al. (2023) [33]	RCT	To establish whether immersive VR was worth considering as a form of physical therapy and to assess the advisability of applying it to restore post-stroke hand function impairment.	First episode, maximum of 12 months since stroke diagnosis, age between 40 and 64 years, acquired motor impairment of the hemiplegic UL, functional brain damage specified with Rankin scale 1–4 at the last hospital discharge. Exclusion criteria: requirement for constant, intensive medical surveillance, presence of active comorbidities, severe arterial or pulmonary hypertension, uncontrolled diabetes, or epilepsy. EG: (n = 10) age 54.9 ± 3.98 years; CG: (n = 10) age 59.2 ± 4.34 years.	18 days, 6 days/wk for 3 consecutive wks for both groups. EG = UL PT using the SciMed system, which incorporates the immersive VR application Virtual Mirror Hand 1.0 with the Oculus Quest 2 VR glasses module. CG = classic mirror therapy treatment relying on a reflection seen in the mirror to facilitate UL rehabilitation.	FMAUE SF-36	T0: baseline T1: post-treatment (3 wks)	No differences between the two treatments were observed; however, patients undergoing VR therapy reported improvements in pain and multiple subjective sensations. The VR application proves to be intuitive, easily understandable, and accessible from the outset, and it was well received and well tolerated by all participants.
3	Chatterjee et al. (2022) [25]	RCT	To assess the effects of a custom-built VR environment on cognitive function, ADL recovery, and length of hospital stay in patients with subacute stroke.	Subjects aged ≥18 with unilateral, confirmed stroke (1 day to 3 weeks prior) leading to cognitive impairment. Exclusions: bilateral weakness, dementia, epilepsy, visual acuity < 6/60, or deemed unfit by therapists. Initial protocol excluded patients with (mRS) > 3; EG (n = 30, age: 77.5 ± 13.5, 13 F, 17 M): VR (a serious game with an explicit program for cognitive rehabilitation group) + UC. CG (n = 10, age: 63.5 ± 26.5, 6 F, 4 M): sham VR + UC	UC: PT, OT, SLT (duration not declared). EG: VR: VIRTUE system: customized 3D immersive VR environment reproducing ADLs (cooking, performing house chores, etc.)—5 days per wk up to 2 wks before hospital discharge—duration not declared. Hardware: HMDs (Oculus Rift S). CG: sessions of VR similar to those of the VIRTUE group (a simple task of picking up object with a hand-held controller)—duration not declared.	MoCA At T0 and T2: NEADL HADS EuroQoLS	T0: baseline T1: post-intervention T2: follow-up, 3 months from T1	This novel immersive VR system had the potential to assist patients with severe cognitive impairment, shorten hospital stays, and supplement the traditional rehabilitation therapy offered by skilled neurotherapy professionals.
4	Hsu et al. (2022) [27]	RCT	To investigate the differences in the effects of using COT, MT, and VR-MT training on the sensorimotor function of the UL in chronic stroke patients.	Subjects with unilateral cerebral infarction/hemorrhage, >6 months post-stroke, FM-UE: 23–60, MMSE score ≥ 24. Exclusion criteria: presence of neglect, impaired vision, severe aphasia. Three randomized groups: COT (n = 17), age 56.9 ± 13.0, 12 F, 5 M; MT (n = 17), age 56.7 ± 11.5, 7 M, 10 F; VR-MT (n = 18), age 52.9 ± 11.8, 8 M, 10 F.	20′ of UC for every group + 30′ of the specific intervention (VR-MT, COT, or MT), twice a wk for 9 wks. All participants received OT + PT and SLT if needed. COT = sensorimotor stimulation and skill training for ADLs. MT = mirror therapy, mirror box. VR-MT = personal computer + Leap Motion Controller (LMC)+ Oculus Rift VR headset.	FMA-UE MAL BBT SWM mAS	T0: baseline T1: post-intervention (9 wks)	VR-MT seemed to have potential effects on restoring upper-extremity motor function for chronic stroke patients.
5	Huang et al. (2022) [28]	RCT	To identify the effects of immersive VR on inflammation, oxidative stress, neuroplasticity, and UL motor function in stroke patients.	Subjects between 20 and 75 yo, stroke onset > 3 months, Brunnstrom stage > 3, diagnosis with computerized tomography or MRI scans, MMSE > 18. Exclusion criteria: participation in other rehabilitation-related or clinical trials within 3 months of the experiment, sensory apraxia, severe impairments in vision or visual perception, receiving warfarin or vitamin K antagonist treatment, high risk of epilepsy, or refusal to undergo the blood test. EG: Immersive VR training group (n = 15), age 50.80 ± 12.32, 9 F, 6 M; CG: COT group (n = 15), age 58.33 ± 11.22.	16 intervention sessions for 60′/day, 2 to 3 days/wk, while attending regular OT. 6–10 tasks were assigned in each session. COT: UL training with peg board, climbing ladder, and stacking cones. VR: VR UL activities based on twenty VR scenes from commercial games. UL movements in most scenes involved aiming, shooting, hitting, waving arms, punching, and throwing objects. VR headset by HTC VIVE was utilized, consisting of an HMD device, two controllers, and two infrared laser emitter units.	FMA-UE AROM SSQ User experience ad hoc questionnaires; serum biomarkers.	T0: baseline T1: post-intervention, 60 days	HO-1, 8-OHdG, and BDNF might be potential serum biomarkers for VR-based interventions in chronic stroke patients.
6	Choi et al. (2021) [26]	RCT	To investigate the effects of VR-based digital practice program on USN rehabilitation in patients with subacute stroke.	Individuals recruited 1–6 months post-stroke, MMSE score ≥ 24; exclusion criteria: participation in experimental rehabilitation or drug research, presence of neglect, severely impaired vision, severely impaired sitting posture, limited neck ROM, presence of headache or dizziness with HMD use. EG: DP group (n = 12), age 63.00 ± 10.02, 5 M, 7 F. CG: (n = 12), age 61.58 ± 9.99, 6 M, 6 F.	Both groups: 5 × 1 h therapy session per wk for 4 wks; the training was based on established motor learning and neurodevelopmental treatment. EG: DP group: digital practice (Oculus Rift DK2 and Leap Motion devices while performing exercises in a seated position). CG: conventional USN-specific training (structured visual tracking; reading, and writing, drawing, and copying; and puzzles) for 3 × 30′/wk.	Line bisection test CBS mBI MFVP (Vertical Version) Horizontal head movements	T0: baseline T1: post-intervention, 4 wks	The DP group showed significantly greater improvements in the following: - Line bisection test (*p* = 0.020); - MFVP Test Vertical Version (*p* = 0.024); - Horizontal head movement: rotation degree and velocity (*p* = 0.007 and *p* = 0.001, respectively).
7	Lin et al. (2021) [29]	RCT	To develop a VR-MT system and to analyze the performance of the proposed system.	Chronic (>6 months) stroke survivors with unilateral cerebral infarction or hemorrhage; FMAUE motor assessment: 23–60; MMSE ≥ 24. Exclusion criteria: Wernicke’s or global aphasia. EG: VR-MT group (n = 9), age 49.7 ± 13.4, 7 M, 2 F. CG: MT group (n = 9), age 58.8 ± 9.6, 6 M, 3 F.	Each session lasted 50′, two days a wk for 9 wks. Intensity was equal between groups. CG: MT group: 30′ of traditional MT + 20′ of motor task-specific training. EG: VR-MT group: 30′ of VR-MT + 20′ of regular motor-task-specific training (technology: Leap Motion Controller with a compact USB peripheral device, Oculus Rift VR goggles, In-house VRMT software, developed with Unity).	FMAUE	T0: baseline T1: post-intervention (9 wks)	In general, VR-MT positively affected UL motor function in stroke patients. After training, the total motor score and the hand component of the FMA showed significant differences (*p* < 0.05) between the two groups and within the VR-MT group.
8	Mekbib et al. (2021) [30]	RCT	To create a unique, completely immersive VR rehabilitation protocol using commercially available peripheral VR equipment that could stimulate and activate motor neurons to aid post-stroke recovery, as well as investigate the effect of the immersive VR system compared to COT for upper-extremity therapy in stroke patients.	Adults (>18 yo), ≤3 months from first episode of ischemic or hemorrhagic stroke, with moderate-to-severe UE impairments. Normal hearing and vision, MMSE > 16. EG: VR intervention + OT (n = 12), age 52.17 ± 13.26, 9 M, 3 F; CG: OT (n = 11), age 61.00 ± 7.69, 8 M, 3 F.	EG: 1 h of VR + 1 h of OT per day, 4 days a wk for two wks. (Technology: HMD from https://www.vive.com accessed on 21 December 2023; 2 HTC Vive tracking stations; Leap Motion tracking technology; ALIENWARE high graphics laptop. The rehabilitation environment, MNVR-Rehab, was created using the Unity 3D game engine. VR: reaching, grasping.) CG: 2 h OT per day, 4 days a wk for two wks, focusing on minimizing spasticity and normalizing movement patterns. The OT intervention included daily living activities, balance control, gait training, weight shift, and distal and proximal UE functional movements.	FMA-UE BI rs-fMRI) data	T0: baseline T1: post-treatment (2 wks)	The VR group revealed significant improvements in FMA-UE scores compared to the CG. A VR system could provide extra advantages for upper-extremity therapy in patients receiving OT.
9	Song et al. (2021) [31]	RCT	To determine the effect of a combination of an immersive VR system and bilateral upper-extremity training addressing ADL on UL function and EEG measurements in stroke patients with chronic hemiplegia.	Diagnosis of stroke with hemiplegia persisting for at least 6 months and MMSE-K (Korean) ≥ 24. Exclusion criteria: visual and sensory issues, psychological instability, a history of craniotomy for brain surgery, and concurrent musculoskeletal disease with upper-extremity involvement. EG: VRBAT (n = 5), age 64.20 ± 7.08, 3 M, 2 F. CG: NBAT (n = 5), age 60.00 ± 10.88, 3 M, 2 F.	EG: VRBAT: immersive VR-based bilateral arm training using Tion software (Human IT Solution, Mokpo-si, Republic of Korea). The VR content included ADL tasks, visual-perception-oriented cognitive exercises, and evaluations delivered through Oculus Rift and Rift controller. The interventions lasted 30′ daily, 5 times a wk, for 4 wks (20 sessions), + 1 h conventional rehabilitation per day. CG: similar real-world tasks for bilateral upper-extremity training, with the same intervention frequency and duration, complemented by one hour of conventional rehabilitation per day.	Manual function test (EMG of biceps brachii, triceps brachii, and wrist extensor and flexor muscles on the affected side); Two-Point Discrimination Test; proprioception rest; stereognosis test; UL Muscle Activity (surface EMG signals of biceps brachii, triceps brachii, extensor carpi, and flexor carpi on the affected side); EEG data (only VRBAT)	T0: baseline T1: post-treatment (4 wks)	The following results were obtained in both groups: - Significant improvement in manual function test (*p* < 0.05); - Significant improvement in relative alpha and beta power values for EEG measurements.
10	Ögün et al. (2019) [12]	RCT	To investigate the effectiveness of Leap Motion-based 3D immersive VR in the rehabilitation of upper extremities in patients with ischemic stroke.	Stroke onset between 6 and 24 months; MMSE ≥ 25; MAS < 3; UE and hand Brunnstrom score ≥ 4. Exclusion criteria: secondary neurological diseases, recurrent stroke, reduced or lost visual field, or hemorrhagic stroke. EG: VR group (n = 33), age 61.48 ± 10.92, 28 M, 5 F; CG: (n = 34), age 59.75 ± 8.07, 23 M, 9 F.	Both groups: three 1 h sessions per wk over six wks. EG: VR group: 4 games using a VR device to play task-oriented games with movement of the arm and forearm, focused on grasping and handling objects. (Technology: CG: 45′ of conventional therapy with active exercises reproducing the same movements used in the VR group. 15′ of passive VR without the need for extremity interactions).	FMA-UE ARAT FIM PASS-IADL PASS-BADL	T0: baseline T1: post-treatment (6 wks)	Both groups showed significant differences (*p* < 0.05) after training for all OMs except for the PASS in the CG. The VR group achieved significantly better results in the independent *t*-test (*p* < 0.05) than the CG. VR effectively improves UL function and ADLs, but no improvement in independence has been observed.

Abbreviations: EG: experimental group; CG: control group; VR: virtual reality; ADL: activities of daily living; RCT: randomized controlled trials; MoCA: Montreal Cognitive Assessment test; NEADL: Nottingham Extended ADL; HADS: Hospital Anxiety and Depression Scale; EuroQoLS: European Quality of Life Survey; USN: unilateral spatial neglect; DP: digital practice; CBS: Catherine Bergego Scale; mBI: Modified Barthel Index; MFVP: Motor-Free Visual Perception Test; COT: conventional occupational therapy; MT: mirror therapy; FMA-UE: Fugl-Meyer Motor Assessment for Upper Extremity; MAL: Motor Activity Log; BBT: Box and Block Test; SWM: Semmes–Weinstein Monofilament test; mAS: Modified Ashworth Scale; AROM: active range of motion; FMA: Fugl-Meyer Assessment scale; BI: Barthel Index; ARAT: Action Research Arm Test; FIM: Functional Independence Measure; PASS-IADL: Performance Assessment of Self-Care Skills— Instrumental ADL; PASS-BADL: Performance Assessment of Self-Care Skills—Basic ADL; OMs: outcome measures; EEG: Electroencephalography; VRBAT: VR-Based Bilateral Arm Training group; NBAT: Normal Bilateral Arm Training group; EMG: Electromyogram; SSQ: Simulator Sickness Questionnaire; PT: physiotherapy; SLT: speech and language therapy; OT: occupational therapy; mRS: Modified Rankin Score, ROM: range of motion; wk: week; F: female; M: male; UL: upper limb; UC: usual care; MRI: magnetic resonance imaging; yo: years old; UE: upper extremity; HMD: head-mounted display; FMUE: Fugl-Meyer Assessment for the upper extremity; BI: Barthel Index; RS-fMRI: resting-state functional magnetic resonance imaging; FC: functional connectivity; ADLs: activities of daily living; MRI: magnetic resonance imaging; TR/TE: Time of Repetition/Time of Echo; FOV: Field of View; mFD: Mean Framewise Displacement; FLIRT: FMRIB’s Linear Image Registration Tool; FNIRT: FMRIB’s Nonlinear Image Registration Tool; MNI152: Montreal Neurological Institute 152; MMSE: mini-mental state examination; rs-fMRI: resting-state functional MRI; h: hour; EMG: electromyography.

## Data Availability

Not applicable.

## Supplementary Materials

The following supporting information can be downloaded at: https://www.mdpi.com/article/10.3390/jcm13010146/s1.
