# Supplementary material for: Effects of Immersive Virtual Reality on Upper-Extremity Stroke Rehabilitation: A Systematic Review with Meta-Analysis
Source: J Clin Med. 2023 Dec 27;13(1):146. doi: 10.3390/jcm13010146 (PMC10780020; doi:10.3390/jcm13010146)
Supplement: Supplementary file 1 [file jcm-13-00146-s001.zip › jcm-2725063-supplementary.pdf]

Supplementary Materials S1

PubMed search strategy:

((stroke[Title/Abstract]) OR (post-stroke[Title/Abstract]) OR (Ischemic [Title/Abstract]) OR (haemorrhagic[Title/Abstract]) OR (transient ischemic attack[Title/Abstract]) OR (cryptogenic[Title/Abstract]) OR (stroke[MeSH Terms]) OR (Brain Ischemia[MeSH Terms]) OR (Cerebral Hemorrhage[MeSH Terms]) OR (Intracranial Thrombosis[MeSH Terms])) AND ((virtual reality[Title/Abstract]) OR (VR[Title/Abstract]) OR (Simulated 3D environment[Title/Abstract]) OR (virtual reality exposure[Title/Abstract]) OR (virtual reality exposure therapy[Title/Abstract]) OR (virtual reality therapy[Title/Abstract]) OR (virtual reality[MeSH Terms]) OR (virtual reality exposure therapy[MeSH Terms])) AND ((Physical Therapy Specialty[MeSH Terms]) OR (Physical Therapy Modalities[MeSH Terms]) OR (physiotherapy[Title/Abstract]) OR (rehabilitation[MeSH Terms]) OR (rehabilitation[Title/Abstract]) OR (conventional physiotherapy[Title/Abstract]) OR (conventional rehabilitation[Title/Abstract]) OR (Physical therapy[Title/Abstract]) OR (Conventional physical therapy[Title/Abstract]) OR (Inpatient therapy[Title/Abstract]) OR (stroke rehabilitation[MeSH Terms]) OR (Neurological Rehabilitation[MeSH Terms]) OR (Exercise Therapy[MeSH Terms])) AND ((Hand[MeSH Terms]) OR (wrist[MeSH Terms]) OR (finger) OR (Hand Joints[MeSH Terms) AND (Hand strength[MeSH Terms) OR (hand dexterity) OR (grip) OR (pinch) OR (hand function) OR (motor functioning) OR (sensory functioning) OR (hand strength rehabilitation[MeSH Terms]) OR (Activities of Daily Living) OR (Upper extremity Function) OR (Upper extremity performance) OR (Coordination) OR (Dexterity) OR (Functional Mobility) OR (Pain) OR (pain[MeSH Terms]) OR (pain perception[MeSH Terms]) OR (pain measurement[MeSH Terms]) OR (strength) OR (Physical Functional Performance[MeSH Terms]))

Cochrane Library search strategy:

#1 ("stroke"):ti,ab,kw OR ("post-stroke"):ti,ab,kw OR ("ischemic"):ti,ab,kw OR ("haemorrhagic"):ti,ab,kw OR ("transient ischemic attack"):ti,ab,kw
#2 ("cryptogenic"):ti,ab,kw
#3 MeSH descriptor: [Brain Ischemia] explode all trees.
#4 MeSH descriptor: [Cerebral Hemorrhage] explode all trees
#5 MeSH descriptor: [Intracranial Thrombosis] explode all trees
#6 #1 OR #2 OR #3 OR #4 OR #5
#7 MeSH descriptor: [Physical Therapy Specialty] explode all trees
#8 MeSH descriptor: [Physical Therapy Modalities] explode all trees
#9 MeSH descriptor: [Rehabilitation] explode all trees
#10 MeSH descriptor: [Stroke Rehabilitation] explode all trees
#11 MeSH descriptor: [Neurological Rehabilitation] explode all trees
#12 MeSH descriptor: [Exercise Therapy] explode all trees
#13 ("physiotherapy"):ti,ab,kw OR ("rehabilitation"):ti,ab,kw OR (conventional physiotherapy):ti,ab,kw OR (conventional rehabilitation):ti,ab,kw OR ("physical therapy"):ti,ab,kw
#14 #7 OR #8 OR #9 OR #10 OR #11 OR #12 OR #13
#15 ("immersive") OR ("head mounted")
#16 (HMD)
#17 #15 OR #16
#18 ("goggles")
#19 #17 OR #18
#20 ("head tracking")
#21 #19 OR #20
#22 ("headset")

#23 #21 OR #22
#24 ("virtual reality"):ti,ab,kw OR ("VR"):ti,ab,kw OR ("stimulated 3D environment"):ti,ab,kw OR ("virtual reality exposure"):ti,ab,kw OR ("virtual reality exposure therapy"):ti,ab,kw
#25 ("virtual reality therapy"):ti,ab,kw
#26 MeSH descriptor: [Virtual Reality] explode all trees
#27 MeSH descriptor: [Virtual Reality Exposure Therapy] explode all trees
#28 #24 OR #25 OR #26 OR #27
#29 #23 OR #28
#30 MeSH descriptor: [Hand Strength] explode all trees
#31 ("hand dexterity") OR ("grip") OR ("pinch") OR ("hand function") OR (motor functioning)
#32 ("sensory functioning") OR ("Activities of Daily Living") OR ("Upper extremity Function") OR ("Upper extremity performance") OR ("coordination")
#33 MeSH descriptor: [Hand Strength] explode all trees
#34 ("dexterity") OR ("Functional Mobility") OR ("pain") OR ("strength")
#35 MeSH descriptor: [Pain] explode all trees
#36 MeSH descriptor: [Pain Perception] explode all trees
#37 MeSH descriptor: [Pain Measurement] explode all trees
#38 MeSH descriptor: [Physical Functional Performance] explode all trees
#39 #30 OR #31 OR #32 OR #33 OR #34 OR #35 OR #36 OR #37 OR #38
#40 ("adverse effect")
#41 #39 OR #40
#42 ("side effect")
#43 #41 OR #42
#44 MeSH descriptor: [Patient Acceptance of Health Care] explode all trees
#45 MeSH descriptor: [Patient Acceptance of Health Care] explode all trees
#46 #43 OR #44 OR #45
#47 #6 AND #14 AND #29 AND #46

Embase search strategy:

('stroke' OR 'post-stroke' OR 'Ischemic' OR 'haemorrhagic' OR 'transient ischemic attack' OR 'cryptogenic' OR 'Brain Ischemia' OR 'Cerebral Hemorrhage' OR 'Intracranial Thrombosis') AND ('Physical Therapy Specialty' OR 'Physical Therapy Modalities' OR 'physiotherapy' OR 'rehabilitation' OR 'conventional physiotherapy' OR 'conventional rehabilitation' OR 'Physical therapy' OR 'Conventional physical therapy' OR 'Inpatient therapy' OR 'stroke rehabilitation' OR 'Neurological Rehabilitation' OR 'Exercise Therapy') AND ('immersive' OR 'head-mounted' OR 'HMD' OR 'goggles' OR 'head tracking' OR 'headset' OR 'virtual reality' OR 'VR' OR 'Simulated 3D environment' OR 'virtual reality exposure' OR 'virtual reality exposure therapy' OR 'virtual reality therapy') AND ('Hand strength' OR 'hand dexterity' OR 'grip' OR 'pinch' OR 'hand function' OR 'motor functioning' OR 'sensory functioning' OR 'hand strength rehabilitation' OR 'Activities of Daily Living' OR 'Upper extremity Function' OR 'Upper extremity performance' OR 'Coordination' OR 'Dexterity' OR 'Functional Mobility' OR 'Pain' OR 'pain perception' OR 'pain measurement' OR 'strength' OR 'Physical Functional Performance' OR 'adverse effect*' OR 'side effect*' OR 'Patient Acceptance')

Scopus search strategy:

TITLE-ABS-KEY("stroke" OR "post-stroke" OR "Ischemic" OR "haemorrhagic" OR "transient ischemic attack" OR "cryptogenic" OR "Brain Ischemia" OR "Cerebral Hemorrhage" OR "Intracranial Thrombosis") AND TITLE-ABS-KEY("Physical Therapy Specialty" OR "Physical Therapy Modalities" OR "physiotherapy" OR "rehabilitation" OR "conventional physiotherapy" OR "conventional rehabilitation" OR "Physical therapy" OR "Conventional physical therapy" OR "Inpatient therapy" OR "stroke rehabilitation" OR "Neurological Rehabilitation" OR "Exercise Therapy") AND TITLE-ABS-KEY("immersive" OR "head-mounted" OR "HMD" OR "goggles" OR

"head tracking" OR "headset" OR "virtual reality" OR "VR" OR "Simulated 3D environment" OR "virtual reality exposure" OR "virtual reality exposure therapy" OR "virtual reality therapy") AND TITLE-ABS-KEY("Hand strength" OR "hand dexterity" OR "grip" OR "pinch" OR "hand function" OR "motor functioning" OR "sensory functioning" OR "hand strength rehabilitation" OR "Activities of Daily Living" OR "Upper extremity Function" OR "Upper extremity performance" OR "Coordination" OR "Dexterity" OR "Functional Mobility" OR "Pain" OR "pain perception" OR "pain measurement" OR "strength" OR "Physical Functional Performance" OR "adverse effect*" OR "side effect*" OR "Patient Acceptance")

Web of Science search strategy:

(((ALL=("stroke" OR "post-stroke" OR "Ischemic" OR "haemorrhagic" OR "transient ischemic attack" OR "cryptogenic" OR "Brain Ischemia" OR "Cerebral Hemorrhage" OR "Intracranial Thrombosis")) AND ALL=("Physical Therapy Specialty" OR "Physical Therapy Modalities" OR "physiotherapy" OR "rehabilitation" OR "conventional physiotherapy" OR "conventional rehabilitation" OR "Physical therapy" OR "Conventional physical therapy" OR "Inpatient therapy" OR "stroke rehabilitation" OR "Neurological Rehabilitation" OR "Exercise Therapy")) AND ALL=("immersive" OR "head-mounted" OR "HMD" OR "goggles" OR "head tracking" OR "headset" OR "virtual reality" OR "VR" OR "Simulated 3D environment" OR "virtual reality exposure" OR "virtual reality exposure therapy" OR "virtual reality therapy")) AND ALL=("Hand strength" OR "hand dexterity" OR "grip" OR "pinch" OR "hand function" OR "motor functioning" OR "sensory functioning" OR "hand strength rehabilitation" OR "Activities of Daily Living" OR "Upper extremity Function" OR "Upper extremity performance" OR "Coordination" OR "Dexterity" OR "Functional Mobility" OR "Pain" OR "pain perception" OR "pain measurement" OR "strength" OR "Physical Functional Performance" OR "adverse effect*" OR "side effect*" OR "Patient Acceptance")

Supplementary Materials S2

List of excluded studies:

1. Wang ZR, Wang P, Xing L, Mei LP, Zhao J, Zhang T. Leap Motion-based virtual reality training for improving motor functional recovery of upper limbs and neural reorganization in subacute stroke patients. *Neural Regen Res*. 2017;12(11):1823-1831. doi:10.4103/1673-5374.219043
2. Subramanian SK, Lourenço CB, Chilingaryan G, Sveistrup H, Levin MF. Arm motor recovery using a virtual reality intervention in chronic stroke: randomized control trial. *Neurorehabil Neural Repair*. 2013;27(1):13-23. doi:10.1177/1545968312449695
3. Lin M, Huang J, Fu J, Sun Y, Fang Q. A VR-Based Motor Imagery Training System With EMG-Based Real-Time Feedback for Post-Stroke Rehabilitation. *IEEE Trans Neural Syst Rehabil Eng*. 2023;31:1-10. doi:10.1109/TNSRE.2022.3210258
4. Lee S, Kim Y, Lee BH. Effect of Virtual Reality-based Bilateral Upper Extremity Training on Upper Extremity Function after Stroke: A Randomized Controlled Clinical Trial. *Occup Ther Int*. 2016;23(4):357-368. doi:10.1002/oti.1437
5. Lee D, Lee M, Lee K, Song C. Asymmetric training using virtual reality reflection equipment and the enhancement of upper limb function in stroke patients: a randomized controlled trial. J Stroke Cerebrovasc Dis. 2014;23(6):1319-1326. doi:10.1016/j.jstrokecerebrovasdis.2013.11.006
6. Kwon JS, Park MJ, Yoon IJ, Park SH. Effects of virtual reality on upper extremity function and activities of daily living performance in acute stroke: a double-blind randomized clinical trial. NeuroRehabilitation. 2012;31(4):379-385. doi:10.3233/NRE-2012-00807
7. Kiper P, Agostini M, Luque-Moreno C, Tonin P, Turolla A. Reinforced feedback in virtual environment for rehabilitation of upper extremity dysfunction after stroke: preliminary data from a randomized controlled trial. Biomed Res Int. 2014;2014:752128. doi:10.1155/2014/752128
8. Faria AL, Andrade A, Soares L, I Badia SB. Benefits of virtual reality based cognitive rehabilitation through simulated activities of daily living: a randomized controlled trial with stroke patients. *J Neuroeng Rehabil*. 2016;13(1):96. Published 2016 Nov 2. doi:10.1186/s12984-016-0204-z

9. Brunner I, Skouen JS, Hofstad H, et al. Virtual Reality Training for Upper Extremity in Subacute Stroke (VIRTUES): A multicenter RCT. *Neurology*. 2017;89(24):2413-2421. doi:10.1212/WNL.0000000000004744
10. Pouplin S, Bonnyaud C, Bouchigny S, et al. Feasibility of a serious game system including a tangible object for post stroke upper limb rehabilitation: a pilot randomized clinical study. *Front Neurol*. 2023;14:1176071. Published 2023 Jun 9. doi:10.3389/fneur.2023.1176071
11. Shin JH, Kim M, Lee JY, Kim MY, Jeon YJ, Kim K. Feasibility of hemispatial neglect rehabilitation with virtual reality-based visual exploration therapy among patients with stroke: randomised controlled trial. *Front Neurosci*. 2023;17:1142663. Published 2023 Apr 20. doi:10.3389/fnins.2023.1142663
12. Rodríguez-Hernández M, Polonio-López B, Corregidor-Sánchez AI, Martín-Conty JL, Mohedano-Moriano A, Criado-Álvarez JJ. Can specific virtual reality combined with conventional rehabilitation improve poststroke hand motor function? A randomized clinical trial. *J Neuroeng Rehabil*. 2023;20(1):38. Published 2023 Apr 4. doi:10.1186/s12984-023-01170-3
13. Shin S, Lee HJ, Chang WH, Ko SH, Shin YI, Kim YH. A Smart Glove Digital System Promotes Restoration of Upper Limb Motor Function and Enhances Cortical Hemodynamic Changes in Subacute Stroke Patients with Mild to Moderate Weakness: A Randomized Controlled Trial. *J Clin Med*. 2022;11(24):7343. Published 2022 Dec 10. doi:10.3390/jcm11247343
14. Cinakli H, Yetisgin A, Sen Dokumaci D, Boyaci A. Effects of adding interactive videogames to conventional rehabilitation program on radiological progression and upper extremity motor function in patients with hemiplegic stroke: a preliminary study [published online ahead of print, 2023 Apr 5]. *Somatosens Mot Res*. 2023;1-9. doi:10.1080/08990220.2023.2194401
15. Chen J, Or CK, Li Z, Yeung EHK, Zhou Y, Hao T. Effectiveness, safety and patients' perceptions of an immersive virtual reality-based exercise system for poststroke upper limb motor rehabilitation: A proof-of-concept and feasibility randomized controlled trial. Digit Health. 2023;9:20552076231203599. Published 2023 Sep 26. doi:10.1177/20552076231203599

Supplementary Materials S3. Funnel plots.

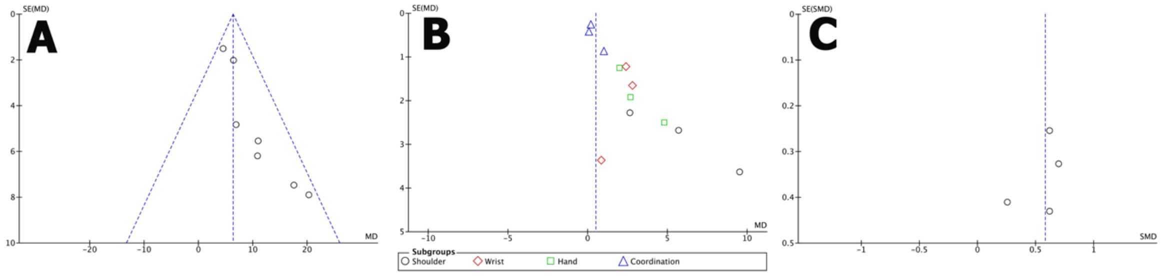

Figure S1. Funnel plot for total FMA-UE results (A), FMA-UE subscale results (B), and ADL results (C).

Table S1. Version 2 of the Cochrane risk-of-bias tool for randomized trials intention-to-treat analysis.

**Unique ID**	Hsu et al. (2022)	**Study ID**	Hsu et al. (2022)	**Assessor**	
**Ref or Label**	Hsu et al., 2022	**Aim**	Assignment to intervention (the 'intention-to-treat' effect)		
**Experimental**		**Comparator**		**Source**	Company-owned trial registry record (e.g., GSK Clinical Study Register record); Research ethics application
**Outcome**		**Results**		**Weight**	1

**Domain**	**Signalling question**	**Response**	**Comments**
**Bias arising from the randomization process**	1.1 Was the allocation sequence random?	Y	Eligible patients were randomly allocated using opaque envelopes with computer-generated random numbers
	1.2 Was the allocation sequence concealed until participants were enrolled and assigned to interventions?	Y	
	1.3 Did baseline differences between intervention groups suggest a problem with the randomization process?	N	No significant differences were found between the groups for any outcome measures before the training.
	**Risk of bias judgement**	**Low**	Eligible patients were randomly allocated using opaque envelopes with computer-generated random numbers. No significant differences were found between the groups for any outcome measures before the training.
**Bias due to deviations from intended interventions**	2.1. Were participants aware of their assigned intervention during the trial?	Y	
	2.2. Were carers and people delivering the interventions aware of participants' assigned intervention during the trial?	Y	
	2.3. If Y/PY/NI to 2.1 or 2.2: Were there deviations from the intended intervention that arose because of the experimental context?	N	2 out of 54 participants dropped out during the trial
	2.4 If Y/PY to 2.3: Were these deviations likely to have affected the outcome?	NA	
	2.5. If Y/PY/NI to 2.4: Were these deviations from intended intervention balanced between groups?	NA	
	2.6 Was an appropriate analysis used to estimate the effect of assignment to intervention?	PY	Modified intention-to-treat (mITT)
	2.7 If N/PN/NI to 2.6: Was there potential for a substantial impact (on the result) of the failure to analyse participants in the group to which they were randomized?	NA	
	**Risk of bias judgement**	**Low**	2 out of 54 participants dropped out during the trial. modified intention-to-treat (mITT)
**Bias due to missing outcome data**	3.1 Were data for this outcome available for all, or nearly all, participants randomized?	PY	
	3.2 If N/PN/NI to 3.1: Is there evidence that result was not biased by missing outcome data?	NA	

	3.3 If N/PN to 3.2: Could missingness in the outcome depend on its true value?	NA	
	3.4 If Y/PY/NI to 3.3: Is it likely that missingness in the outcome depended on its true value?	NA	
	**Risk of bias judgement**	**Low**	
**Bias in measurement of the outcome**	4.1 Was the method of measuring the outcome inappropriate?	N	
	4.2 Could measurement or ascertainment of the outcome have differed between intervention groups?	N	
	4.3 Were outcome assessors aware of the intervention received by study participants?	N	Two evaluators were blinded to the participant's condition.
	4.4 If Y/PY/NI to 4.3: Could assessment of the outcome have been influenced by knowledge of intervention received?	NA	
	4.5 If Y/PY/NI to 4.4: Is it likely that assessment of the outcome was influenced by knowledge of intervention received?	NA	
	**Risk of bias judgement**	**Low**	Two evaluators were blinded to the participant's condition.
**Bias in selection of the reported result**	5.1 Were the data that produced this result analysed in accordance with a pre-specified analysis plan that was finalized before unblinded outcome data were available for analysis?	Y	Registration: NCT03329417
	5.2 ... multiple eligible outcome measurements (e.g., scales, definitions, time points) within the outcome domain?	N	
	5.3 ... multiple eligible analyses of the data?	N	
	**Risk of bias judgement**	**Low**	Registration: NCT03329417
**Overall bias**	**Risk of bias judgement**	**Low**	Eligible patients were randomly allocated using opaque envelopes with computer-generated random numbers. No significant differences were found between the groups for any outcome measures before the training.2 out of 54 participants dropped out during the trial modified intention-to-treat (mITT). Two evaluators were blinded to the participant's condition. registration: NCT03329417

**Unique ID**	Huang et al. (2022)	**Study ID**	Huang et al. (2022)	**Assessor**	
**Ref or Label**	Chien-Yu et al.,	**Aim**	Assignment to intervention (the 'intention-to-treat' effect)		
**Experimental**		**Comparator**		**Source**	Regulatory document (e.g., Clinical Study Report, Drug Approval Package)
**Outcome**		**Results**		**Weight**	1

Domain	Signalling question	Response	Comments
	1.1 Was the allocation sequence random?	Y	

**Bias arising from the randomization process**	1.2 Was the allocation sequence concealed until participants were enrolled and assigned to interventions?	Y	Randomization procedures were performed using a randomization table generated online.<br>The allocation sequence was attained by a researcher not involved in the intervention and evaluation.
	1.3 Did baseline differences between intervention groups suggest a problem with the randomization process?	N	No significant differences were found between the groups. for any outcome measures before the training.
	**Risk of bias judgement**	**Low**	Randomization procedures were performed using a randomization table generated online.<br>the allocation sequence was attained by a researcher not involved in the intervention and evaluation. No significant differences were found between the groups for any outcome measures before the training.
**Bias due to deviations from intended interventions**	2.1. Were participants aware of their assigned intervention during the trial?	Y	Awareness of participants to the assigned intervention depends on the nature of the intervention. Assessor were blinded
	2.2. Were carers and people delivering the interventions aware of participants' assigned intervention during the trial?	Y	
	2.3. If Y/PY/NI to 2.1 or 2.2: Were there deviations from the intended intervention that arose because of the experimental context?	N	
	2.4 If Y/PY to 2.3: Were these deviations likely to have affected the outcome?	NA	
	2.5. If Y/PY/NI to 2.4: Were these deviations from intended intervention balanced between groups?	NA	
	2.6 Was an appropriate analysis used to estimate the effect of assignment to intervention?	Y	Effect was measured using two-way mixed analyses of variance (ANOVAs)
	2.7 If N/PN/NI to 2.6: Was there potential for a substantial impact (on the result) of the failure to analyse participants in the group to which they were randomized?	NA	
	**Risk of bias judgement**	**Low**	Awareness of participants to the assigned intervention depends on the nature of the intervention. Assessor were blinded. Effect was measured using two-way mixed analyses of variance (ANOVAs)
**Bias due to missing outcome data**	3.1 Were data for this outcome available for all, or nearly all, participants randomized?	Y	No missing data
	3.2 If N/PN/NI to 3.1: Is there evidence that result was not biased by missing outcome data?	NA	
	3.3 If N/PN to 3.2: Could missingness in the outcome depend on its true value?	NA	
	3.4 If Y/PY/NI to 3.3: Is it likely that missingness in the outcome depended on its true value?	NA	
	**Risk of bias judgement**	**Low**	No missing data
**Bias in measurement of the outcome**	4.1 Was the method of measuring the outcome inappropriate?	N	Method is appropriate
	4.2 Could measurement or ascertainment of the outcome have differed between intervention groups?	N	Same outcome measure between groups

	4.3 Were outcome assessors aware of the intervention received by study participants?	N	Assessor was blinded
	4.4 If Y/PY/NI to 4.3: Could assessment of the outcome have been influenced by knowledge of intervention received?	NA	
	4.5 If Y/PY/NI to 4.4: Is it likely that assessment of the outcome was influenced by knowledge of intervention received?	NA	
	**Risk of bias judgement**	**Low**	Method is appropriate. Same outcome measure between groups. Assessor was blinded
**Bias in selection of the reported result**	5.1 Were the data that produced this result analysed in accordance with a pre-specified analysis plan that was finalized before unblinded outcome data were available for analysis?	Y	Protocol registration (chiCTR2100047853)
	5.2 ... multiple eligible outcome measurements (e.g., scales, definitions, time points) within the outcome domain?	N	
	5.3 ... multiple eligible analyses of the data?	N	
	**Risk of bias judgement**	**Low**	Protocol registration (chiCTR2100047853)
**Overall bias**	**Risk of bias judgement**	**Low**	Randomization procedures were performed using a randomization table generated online. The allocation sequence was attained by a researcher not involved in the intervention and evaluation. No significant differences were found between the group for any outcome measures before the training. Awareness of participants to the assigned intervention depends on the nature of the intervention Effect was measured using two-way mixed analyses of variance (ANOVAs)<br>No missing data. Method is appropriate. Same outcome measure between groups. Assessor was blinded; Protocol registration (chiCTR2100047853)

**Unique ID**	Chatterjee et al. (2022)	**Study ID**	Chatterjee et al. (2022)	**Assessor**	
**Ref or Label**		**Aim**	Assignment to intervention (the 'intention-to-treat' effect)		
**Experimental**		**Comparator**		**Source**	
**Outcome**		**Results**		**Weight**	1

**Domain**	**Signalling question**	**Respons e**	**Comments**
**Bias arising from the randomization process**	1.1 Was the allocation sequence random?	Y	Quote "Block randomization with block sizes of 4 and 8". Quote "Researchers undertaking recruitment and randomization had no prior knowledge or involvement in the generation of randomization lists."
	1.2 Was the allocation sequence concealed until participants were enrolled and assigned to interventions?	Y	
	1.3 Did baseline differences between intervention groups suggest a problem with the randomization process?	PN	No substantial differences between intervention group sizes, compared with the intended allocation ratio (3:1). No significant difference except for age.
	**Risk of bias judgement**	**Low**	Quote "Block randomization with block sizes of 4 and 8". Quote "Researchers undertaking recruitment and randomization had no prior knowledge or involvement in the generation of randomization lists.". No substantial differences between intervention group sizes, compared with the intended allocation ratio (3:1) except for age.

**Bias due to deviations from intended interventions**	2.1. Were participants aware of their assigned intervention during the trial?	PY	Depending on the type of intervention, participants, caregivers, and therapists may be aware of it.
	2.2.Were carers and people delivering the interventions aware of participants' assigned intervention during the trial?	PY	
	2.3. If Y/PY/NI to 2.1 or 2.2: Were there deviations from the intended intervention that arose because of the experimental context?	PY	Quote: "This is only to be expected given the sample population but has impacted the sample size used in the statistical analysis, particularly the size of the sham group."
	2.4 If Y/PY to 2.3: Were these deviations likely to have affected the outcome?	PY	Quote: "The original intention was to also use CAM but as the majority of the participants were either getting tired or refusing to complete the CAM examination" – CAM was removed
	2.5. If Y/PY/NI to 2.4: Were these deviations from intended intervention balanced between groups?	N	Especially SHAM group
	2.6 Was an appropriate analysis used to estimate the effect of assignment to intervention?	Y	Nonparametric one-way ANOVA
	2.7 If N/PN/NI to 2.6: Was there potential for a substantial impact (on the result) of the failure to analyse participants in the group to which they were randomized?	NA	
	**Risk of bias judgement**	**High**	Depending on the type of intervention, participants, caregivers, and therapists may be aware of it. Quote : "This is only to be expected given the sample population but has impacted the sample size used in the statistical analysis, particularly the size of the sham group.". Quote: "The original intention was to also use CAM but as the majority of the participants were either getting tired or refusing to complete the CAM examination" – CAM was removed. Especially SHAM group. non parametric one-way ANOVA
**Bias due to missing outcome data**	3.1 Were data for this outcome available for all, or nearly all, participants randomized?	N	14/40 missing data
	3.2 If N/PN/NI to 3.1: Is there evidence that result was not biased by missing outcome data?	Y	Statistically correct bias "A Shapiro-Wilk test confirmed that this data is not normally distributed in all three groups and so a non-parametric one-way ANOVA (the Kruskal-Wallis test) was applied"
	3.3 If N/PN to 3.2: Could missingness in the outcome depend on its true value?	NA	
	3.4 If Y/PY/NI to 3.3: Is it likely that missingness in the outcome depended on its true value?	NA	
	**Risk of bias judgement**	**Low**	14/40 missing data. Statistically correct bias "A Shapiro-Wilk test confirmed that this data is not normally distributed in all three groups and so a non-parametric one-way ANOVA (the Kruskal-Wallis test) was applied"
**Bias in measurement of the outcome**	4.1 Was the method of measuring the outcome inappropriate?	N	Methods are appropriate
	4.2 Could measurement or ascertainment of the outcome have differed between intervention groups?	PN	Measurement between groups should have been the same
	4.3 Were outcome assessors aware of the intervention received by study participants?	N	Blinded assessor
	4.4 If Y/PY/NI to 4.3: Could assessment of the outcome have been influenced by knowledge of intervention received?	NA	

	4.5 If Y/PY/NI to 4.4: Is it likely that assessment of the outcome was influenced by knowledge of intervention received?	NA	
	**Risk of bias judgement**	**Low**	Methods are appropriate. Measurement between groups should have been the same. Blinded assessor
**Bias in selection of the reported result**	5.1 Were the data that produced this result analysed in accordance with a pre-specified analysis plan that was finalized before unblinded outcome data were available for analysis?	Y	Approval of all ethical and experimental procedures and protocols was granted by the Northwest-Liverpool Central Research Ethics Committee under Reference No. 19/NW/0419.
	5.2 ... multiple eligible outcome measurements (e.g. scales, definitions, time points) within the outcome domain?	N	See table III
	5.3 ... multiple eligible analyses of the data?	N	See table III
	**Risk of bias judgement**	**Low**	Approval of all ethical and experimental procedures and protocols was granted by the Northwest-Liverpool Central Research Ethics Committee under Reference No. 19/NW/0419. See table III
**Overall bias**	**Risk of bias judgement**	**High**	Quote "Block randomization with block sizes of 4 and 8". Quote "Researchers undertaking recruitment and randomization had no prior knowledge or involvement in the generation of randomization lists.". No substantial differences between intervention group sizes, compared with the intended allocation ratio (3:1). No significant difference except for age. Depending on the type of intervention, participants, caregivers, and therapists may be aware of it. Quote: "This is only to be expected given the sample population but has impacted the sample size used in the statistical analysis, particularly the size of the sham group.". Quote: "The original intention was to also use CAM but as the majority of the participants were either getting tired or refusing to complete the CAM examination" – CAM was removed. Especially SHAM group. nonparametric one-way ANOVA. 14/40 missing data. Statistically correct bias "A Shapiro-Wilk test confirmed that this data is not normally distributed in all three groups and so a non-parametric one-way ANOVA (the Kruskal-Wallis test) was applied". Methods are appropriate. Measurement between groups should have been the same. Blinded assessor. Approval of all ethical and experimental procedures and protocols was granted by the Northwest-Liverpool Central Research Ethics Committee under Reference No. 19/NW/0419. See table III

**Unique ID**	Choi et al. (2021)	**Study ID**	Choi et al. (2021)	**Assessor**	
**Ref or Label**		**Aim**	Assignment to intervention (the 'intention-to-treat' effect)		
**Experimental**		**Comparator**		**Source**	
**Outcome**		**Results**		**Weight**	1

Domain	Signalling question	Response	Comments
**Bias arising from the randomization process**	1.1 Was the allocation sequence random?	Y	Quote: "opaque closed envelope". Quote: "Participants were randomized into 2 groups by a therapist not involved in the study: a digital practice group and a control group"
	1.2 Was the allocation sequence concealed until participants were enrolled and assigned to interventions?	Y	
	1.3 Did baseline differences between intervention groups suggest a problem with the randomization process?	N	No significant differences
	**Risk of bias judgement**	**Low**	Quote: "opaque closed envelope". Quote: "Participants were randomized into 2 groups by a therapist not involved in the study: a digital practice group and a control group". No significant differences

Bias due to deviations from intended interventions	2.1.Were participants aware of their assigned intervention during the trial?	PY	Depending on the type of intervention, participants, caregivers, and therapists may be aware of it.
	2.2.Were carers and people delivering the interventions aware of participants' assigned intervention during the trial?	PY	
	2.3. If Y/PY/NI to 2.1 or 2.2: Were there deviations from the intended intervention that arose because of the experimental context?	N	No deviations
	2.4 If Y/PY to 2.3: Were these deviations likely to have affected the outcome?	NA	
	2.5. If Y/PY/NI to 2.4: Were these deviations from intended intervention balanced between groups?	NA	
	2.6 Was an appropriate analysis used to estimate the effect of assignment to intervention?	Y	
	2.7 If N/PN/NI to 2.6: Was there potential for a substantial impact (on the result) of the failure to analyse participants in the group to which they were randomized?	NA	
	**Risk of bias judgement**	**Low**	Depending on the type of intervention, participants, caregivers, and therapists may be aware of it. No deviations
Bias due to missing outcome data	3.1 Were data for this outcome available for all, or nearly all, participants randomized?	Y	No missing data
	3.2 If N/PN/NI to 3.1: Is there evidence that result was not biased by missing outcome data?	NA	
	3.3 If N/PN to 3.2: Could missingness in the outcome depend on its true value?	NA	
	3.4 If Y/PY/NI to 3.3: Is it likely that missingness in the outcome depended on its true value?	NA	
	**Risk of bias judgement**	**Low**	No missing data
Bias in measurement of the outcome	4.1 Was the method of measuring the outcome inappropriate?	N	The methods for evaluating outcome were appropriate for determining if the desired result had been achieved.
	4.2 Could measurement or ascertainment of the outcome have differed between intervention groups?	N	Same methods between groups
	4.3 Were outcome assessors aware of the intervention received by study participants?	N	Quote "Evaluators who do not know group assignments have experience with testing and are eligible."
	4.4 If Y/PY/NI to 4.3: Could assessment of the outcome have been influenced by knowledge of intervention received?	NA	
	4.5 If Y/PY/NI to 4.4: Is it likely that assessment of the outcome was influenced by knowledge of intervention received?	NA	

	Risk of bias judgement		Low	The methods for evaluating outcome were appropriate for determining if the desired result had been achieved. Same methods between groups. Quote "Evaluators who do not know group assignments have experience with testing and are eligible."	
Bias in selection of the reported result	5.1 Were the data that produced this result analysed in accordance with a pre-specified analysis plan that was finalized before unblinded outcome data were available for analysis?		NI	The article did not report the registration of the trial	
	5.2 ... multiple eligible outcome measurements (e.g. scales, definitions, time points) within the outcome domain?		N	Table 2, 3, 4	
	5.3 ... multiple eligible analyses of the data?		N		
	Risk of bias judgement		Some concerns	The paper did not report the registration of the trial. Table 2, 3, 4	
Overall bias	Risk of bias judgement		Some concerns	Quote: "opaque closed envelope". Quote: "Participants were randomized into 2 groups by a therapist not involved in the study: a digital practice group and a control group". No significant differences. Depending on the type of intervention, participants, caregivers, and therapists may be aware of it. No deviations and No missing data. The methods for evaluating outcome were appropriate for determining if the desired result had been achieved. Same methods between groups. Quote "Evaluators who do not know group assignments have experience with testing and are eligible.". The paper did not report the registration of the trial. Table 2, 3, 4.	
**Unique ID**	Lin et al. (2021)	**Study ID**	Lin et al. (2021)	**Assessor**	
**Ref or Label**	Lin et al., 2020	**Aim**	Assignment to intervention (the 'intention-to-treat' effect)		
**Experimental**		**Comparator**		**Source**	Research ethics application
**Outcome**		**Results**		**Weight**	1
**Domain**	**Signalling question**		**Response**	**Comments**	
Bias arising from the randomization process	1.1 Was the allocation sequence random?		Y	Randomly allocated using opaque envelopes with computer-generated random numbers	
	1.2 Was the allocation sequence concealed until participants were enrolled and assigned to interventions?		Y		
	1.3 Did baseline differences between intervention groups suggest a problem with the randomization process?		N	No significant differences were found between the groups for any outcome measures before the training.	
	Risk of bias judgement		Low	Randomly allocated using opaque envelopes with computer-generated random numbers. No significant differences were found between the groups for any outcome measures before the training.	
Bias due to deviations from intended interventions	2.1.Were participants aware of their assigned intervention during the trial?		Y		
	2.2.Were carers and people delivering the interventions aware of participants' assigned intervention during the trial?		Y		
	2.3. If Y/PY/NI to 2.1 or 2.2: Were there deviations from the intended intervention that arose because of the experimental context?		N	2 out of 54 participants dropped out during the trial	

	2.4 If Y/PY to 2.3: Were these deviations likely to have affected the outcome?	NA	
	2.5. If Y/PY/NI to 2.4: Were these deviations from intended intervention balanced between groups?	NA	
	2.6 Was an appropriate analysis used to estimate the effect of assignment to intervention?	Y	
	2.7 If N/PN/NI to 2.6: Was there potential for a substantial impact (on the result) of the failure to analyse participants in the group to which they were randomized?	NA	
	**Risk of bias judgement**	**Low**	2 out of 54 participants dropped out during the trial
**Bias due to missing outcome data**	3.1 Were data for this outcome available for all, or nearly all, participants randomized?	Y	
	3.2 If N/PN/NI to 3.1: Is there evidence that result was not biased by missing outcome data?	NA	
	3.3 If N/PN to 3.2: Could missingness in the outcome depend on its true value?	NA	
	3.4 If Y/PY/NI to 3.3: Is it likely that missingness in the outcome depended on its true value?	NA	
	**Risk of bias judgement**	**Low**	
**Bias in measurement of the outcome**	4.1 Was the method of measuring the outcome inappropriate?	N	
	4.2 Could measurement or ascertainment of the outcome have differed between intervention groups?	N	
	4.3 Were outcome assessors aware of the intervention received by study participants?	N	Two external evaluators blinded to the participant's condition were employed.
	4.4 If Y/PY/NI to 4.3: Could assessment of the outcome have been influenced by knowledge of intervention received?	NA	
	4.5 If Y/PY/NI to 4.4: Is it likely that assessment of the outcome was influenced by knowledge of intervention received?	NA	
	**Risk of bias judgement**	**Low**	Two external evaluators blinded to the participant's condition were employed.
**Bias in selection of the reported result**	5.1 Were the data that produced this result analysed in accordance with a pre-specified analysis plan that was finalized before unblinded outcome data were available for analysis?	NI	
	5.2 ... multiple eligible outcome measurements (e.g., scales, definitions, time points) within the outcome domain?	NI	
	5.3 ... multiple eligible analyses of the data?	NI	
	**Risk of bias judgement**	**Some concerns**	There was no registration of the trial, making hard to know if results were in accordance with a pre-specified plan.

**Overall bias**	**Risk of bias judgement**	**Some concerns**	Randomly allocated using opaque envelopes with computer-generated random numbers. No significant differences were found between the groups for any outcome measures before the training.2 out of 54 participants dropped out during the trial. Two external evaluators blinded to the participant's condition were employed. There was no registration of the trial, making hard to know if results were in accordance with a pre-specified plan.

**Unique ID**	Mekbib et al. (2021)	**Study ID**	Mekbib et al. (2021)	**Assessor**	
**Ref or Label**		**Aim**	Assignment to intervention (the 'intention-to-treat' effect)		
**Experimental**		**Comparator**		**Source**	
**Outcome**		**Results**		**Weight**	1

**Domain**	**Signalling question**	**Response**	**Comments**
**Bias arising from the randomization process**	1.1 Was the allocation sequence random?	Y	Random numbers are generated by a computer program. Physician who was unaware of the study protocol.
	1.2 Was the allocation sequence concealed until participants were enrolled and assigned to interventions?	Y	
	1.3 Did baseline differences between intervention groups suggest a problem with the randomization process?	N	Moderate outliers were detected and removed from further statistical analysis
	**Risk of bias judgement**	**Low**	Random numbers are generated by a computer program. Physician who was unaware of the study protocol. Moderate outliers were detected and removed from further statistical analysis
**Bias due to deviations from intended interventions**	2.1.Were participants aware of their assigned intervention during the trial?	Y	Awareness of participants and therapist about the assigned intervention
	2.2.Were carers and people delivering the interventions aware of participants' assigned intervention during the trial?	Y	
	2.3. If Y/PY/NI to 2.1 or 2.2: Were there deviations from the intended intervention that arose because of the experimental context?	N	No deviations from intended intervention found
	2.4 If Y/PY to 2.3: Were these deviations likely to have affected the outcome?	NA	
	2.5. If Y/PY/NI to 2.4: Were these deviations from intended intervention balanced between groups?	NA	
	2.6 Was an appropriate analysis used to estimate the effect of assignment to intervention?	Y	Quote: "Demographic information, clinical outcomes, and other statistics were analysed using the SPSS software package (version 22, IBM). The chi-square analysis and Fisher's exact test were used to assess the significance of differences between categorical variables."
	2.7 If N/PN/NI to 2.6: Was there potential for a substantial impact (on the result) of the failure to analyse participants in the group to which they were randomized?	NA	
	**Risk of bias judgement**	**Low**	Awareness of participants and therapist about the assigned intervention. No deviations from intended intervention found. Quote: "Demographic information, clinical outcomes, and other statistics were analysed using the SPSS software package (version 22, IBM). The chi-square analysis and Fisher's exact test were used to assess the significance of differences between categorical variables."

**Bias due to missing outcome data**	3.1 Were data for this outcome available for all, or nearly all, participants randomized?	Y	No missing data
	3.2 If N/PN/NI to 3.1: Is there evidence that result was not biased by missing outcome data?	NA	
	3.3 If N/PN to 3.2: Could missingness in the outcome depend on its true value?	NA	
	3.4 If Y/PY/NI to 3.3: Is it likely that missingness in the outcome depended on its true value?	NA	
	**Risk of bias judgement**	**Low**	No missing data
**Bias in measurement of the outcome**	4.1 Was the method of measuring the outcome inappropriate?	N	The outcome measuring techniques used were suitable for evaluating the intended outcome.
	4.2 Could measurement or ascertainment of the outcome have differed between intervention groups?	Y	Quote: "The OT group did not participate in the MRI scanning."
	4.3 Were outcome assessors aware of the intervention received by study participants?	NA	
	4.4 If Y/PY/NI to 4.3: Could assessment of the outcome have been influenced by knowledge of intervention received?	NA	
	4.5 If Y/PY/NI to 4.4: Is it likely that assessment of the outcome was influenced by knowledge of intervention received?	NA	
	**Risk of bias judgement**	**High**	The outcome measuring techniques used were suitable for evaluating the intended outcome. Quote: "The OT group did not participate in the MRI scanning."
**Bias in selection of the reported result**	5.1 Were the data that produced this result analysed in accordance with a pre-specified analysis plan that was finalized before unblinded outcome data were available for analysis?	Y	Study protocol was conducted in accordance with the Helsinki Declaration of Ethical Principles for Medical Research Involving Human Subjects. IRB approval was issued by the local Research Ethics Committee (Zhejiang People's Hospital IRB# 2018KY021).
	5.2 ... multiple eligible outcome measurements (e.g. scales, definitions, time points) within the outcome domain?	N	See Table III
	5.3 ... multiple eligible analyses of the data?	N	
	**Risk of bias judgement**	**Low**	Study protocol was conducted in accordance with the Helsinki Declaration of Ethical Principles for Medical Research Involving Human Subjects. IRB approval was issued by the local Research Ethics Committee (Zhejiang People's Hospital IRB# 2018KY021). See Table III
**Overall bias**	**Risk of bias judgement**	**High**	Random numbers are generated by a computer program. Physician who was unaware of the study protocol. Moderate outliers were detected and removed from further statistical analysis. Awareness of participants and therapist about the assigned intervention. No deviations from intended intervention found. Quote: "Demographic information, clinical outcomes, and other statistics were analysed using the SPSS software package (version 22, IBM). The chi-square analysis and Fisher's exact test were used to assess the significance of differences between categorical variables.". No missing data. The outcome measuring techniques used were suitable for evaluating the intended outcome. Quote: "The OT group did not participate in the MRI scanning.". study protocol was conducted in accordance with the Helsinki

			Declaration of Ethical Principles for Medical Research Involving Human Subjects. IRB approval was issued by the local Research Ethics Committee (Zhejiang People's Hospital IRB# 2018KY021). See Table III

**Unique ID**	Song et al. (2021)	**Study ID**	Song et al. (2021)	**Assessor**	
**Ref or Label**		**Aim**	Assignment to intervention (the 'intention-to-treat' effect)		
**Experimental**		**Comparator**		**Source**	
**Outcome**		**Results**		**Weight**	1

**Domain**	**Signalling question**	**Response**	**Comments**
**Bias arising from the randomization process**	1.1 Was the allocation sequence random?	Y	"Answer 'Yes' if envelopes or drug containers were used appropriately. Envelopes should be opaque, sequentially numbered, sealed with a tamper-proof seal and opened only after the envelope has been irreversibly assigned to the participant."
	1.2 Was the allocation sequence concealed until participants were enrolled and assigned to interventions?	Y	
	1.3 Did baseline differences between intervention groups suggest a problem with the randomization process?	N	
	**Risk of bias judgement**	**Low**	"Answer 'Yes' if envelopes or drug containers were used appropriately. Envelopes should be opaque, sequentially numbered, sealed with a tamper-proof seal and opened only after the envelope has been irreversibly assigned to the participant."
**Bias due to deviations from intended interventions**	2.1. Were participants aware of their assigned intervention during the trial?	Y	
	2.2. Were carers and people delivering the interventions aware of participants' assigned intervention during the trial?	Y	
	2.3. If Y/PY/NI to 2.1 or 2.2: Were there deviations from the intended intervention that arose because of the experimental context?	N	
	2.4 If Y/PY to 2.3: Were these deviations likely to have affected the outcome?	NA	
	2.5. If Y/PY/NI to 2.4: Were these deviations from intended intervention balanced between groups?	NA	
	2.6 Was an appropriate analysis used to estimate the effect of assignment to intervention?	Y	
	2.7 If N/PN/NI to 2.6: Was there potential for a substantial impact (on the result) of the failure to analyse participants in the group to which they were randomized?	NA	
	**Risk of bias judgement**	**Low**	
**Bias due to missing outcome data**	3.1 Were data for this outcome available for all, or nearly all, participants randomized?	Y	
	3.2 If N/PN/NI to 3.1: Is there evidence that result was not biased by missing outcome data?	NA	
	3.3 If N/PN to 3.2: Could missingness in the outcome depend on its true value?	NA	
	3.4 If Y/PY/NI to 3.3: Is it likely that missingness in the outcome depended on its true value?	NA	
	**Risk of bias judgement**	**Low**	

Bias in measurement of the outcome	4.1 Was the method of measuring the outcome inappropriate?	N	
	4.2 Could measurement or ascertainment of the outcome have differed between intervention groups?	N	
	4.3 Were outcome assessors aware of the intervention received by study participants?	NI	
	4.4 If Y/PY/NI to 4.3: Could assessment of the outcome have been influenced by knowledge of intervention received?	NI	The assessors could have had an impact on the outcome for instance strength test where the motivation is important
	4.5 If Y/PY/NI to 4.4: Is it likely that assessment of the outcome was influenced by knowledge of intervention received?	NI	
	**Risk of bias judgement**	**High**	The assessors could have had an impact on the outcome for instance strength test where the motivation is important
**Bias in selection of the reported result**	5.1 Were the data that produced this result analysed in accordance with a pre-specified analysis plan that was finalized before unblinded outcome data were available for analysis?	PY	
	5.2 ... multiple eligible outcome measurements (e.g., scales, definitions, time points) within the outcome domain?	N	
	5.3 ... multiple eligible analyses of the data?	N	
	**Risk of bias judgement**	**Low**	
**Overall bias**	**Risk of bias judgement**	**High**	"Answer 'Yes' if envelopes or drug containers were used appropriately. Envelopes should be opaque, sequentially numbered, sealed with a tamper-proof seal and opened only after the envelope has been irreversibly assigned to the participant." The assessors could have had an impact on the outcome for instance strength test where the motivation is important

**Unique ID**	Ögün et al. (2019)	**Study ID**	Ögün et al. (2019)	**Assessor**	
**Ref or Label**	Ogun et all.,	**Aim**	Assignment to intervention (the 'intention-to-treat' effect)		
**Experimental**		**Comparator**		**Source**	
**Outcome**		**Results**		**Weight**	1

Domain	Signalling question	Response	Comments
**Bias arising from the randomization process**	1.1 Was the allocation sequence random?	Y	Patients were randomly divided into two groups, VR or control, with stratified randomization according to age, sex, and stroke onset, using an online randomization website. No significant differences were found between the groups for any outcome measures before the training.
	1.2 Was the allocation sequence concealed until participants were enrolled and assigned to interventions?	Y	
	1.3 Did baseline differences between intervention groups suggest a problem with the randomization process?	N	An online randomization website. No significant differences were found between the groups for any outcome measures before the training.
	**Risk of bias judgement**	**Low**	Patients were randomly divided into two groups, VR or control, with stratified randomization according to age, sex, and stroke onset, using an online randomization website. No significant differences were found between the groups for any outcome measures before the training. An online randomization website.
**Bias due to deviations from**	2.1. Were participants aware of their assigned intervention during the trial?	N	Both patients and outcome assessors were masked, which was achieved by using sham VR therapy with the control group and the outcome assessor being blinded to the groups.

**intended interventions**	2.2. Were carers and people delivering the interventions aware of participants' assigned intervention during the trial?	PY	
	2.3. If Y/PY/NI to 2.1 or 2.2: Were there deviations from the intended intervention that arose because of the experimental context?	PN	During the study, 10 patients from the VR group and nine patients from the control group discontinued their sessions.
	2.4 If Y/PY to 2.3: Were these deviations likely to have affected the outcome?	NA	
	2.5. If Y/PY/NI to 2.4: Were these deviations from intended intervention balanced between groups?	NA	
	2.6 Was an appropriate analysis used to estimate the effect of assignment to intervention?	N	
	2.7 If N/PN/NI to 2.6: Was there potential for a substantial impact (on the result) of the failure to analyse participants in the group to which they were randomized?	PN	
	**Risk of bias judgement**	**Some concerns**	Both patients and outcome assessors were masked, which was achieved by using sham VR therapy with the control group and the outcome assessor being blinded to the groups. During the study, 10 patients from the VR group and nine patients from the control group discontinued their sessions.
**Bias due to missing outcome data**	3.1 Were data for this outcome available for all, or nearly all, participants randomized?	N	
	3.2 If N/PN/NI to 3.1: Is there evidence that result was not biased by missing outcome data?	PY	
	3.3 If N/PN to 3.2: Could missingness in the outcome depend on its true value?	NA	
	3.4 If Y/PY/NI to 3.3: Is it likely that missingness in the outcome depended on its true value?	NA	
	**Risk of bias judgement**	**Low**	
**Bias in measurement of the outcome**	4.1 Was the method of measuring the outcome inappropriate?	N	
	4.2 Could measurement or ascertainment of the outcome have differed between intervention groups?	N	
	4.3 Were outcome assessors aware of the intervention received by study participants?	N	
	4.4 If Y/PY/NI to 4.3: Could assessment of the outcome have been influenced by knowledge of intervention received?	NA	
	4.5 If Y/PY/NI to 4.4: Is it likely that assessment of the outcome was influenced by knowledge of intervention received?	NA	
	**Risk of bias judgement**	**Low**	
**Bias in selection of the reported result**	5.1 Were the data that produced this result analysed in accordance with a pre-specified analysis plan that	Y	The research protocol was registered with ClinicalTrials.gov ID: NCT03135418).

	was finalized before unblinded outcome data were available for analysis?		
	5.2 ... multiple eligible outcome measurements (e.g., scales, definitions, time points) within the outcome domain?	N	
	5.3 ... multiple eligible analyses of the data?	PN	
	**Risk of bias judgement**	**Low**	The research protocol was registered with ClinicalTrials.gov ID: NCT03135418).
**Overall bias**	**Risk of bias judgement**	**Some concerns**	Patients were randomly divided into two groups, VR or control, with stratified randomization according to age, sex and stroke onset, using an online randomization website. No significant differences were found between the groups for any outcome measures before the training. An online randomization website. Both patients and outcome assessors were masked, which was achieved by using sham VR therapy with the control group and the outcome assessor being blinded to the groups. During the study, 10 patients from the VR group and nine patients from the control group discontinued their sessions. The research protocol was registered with ClinicalTrials.gov ID: NCT03135418).

**Unique ID**	Sip et al. (2023)	**Study ID**	Sip et al. (2023)	**Assessor**	
**Ref or Label**	Sip et al. (2023)	**Aim**	Assignment to intervention (the 'intention-to-treat' effect)		
**Experimental**		**Comparator**		**Source**	
**Outcome**		**Results**		**Weight**	1

Domain	Signalling question	Response	Comments
**Bias arising from the randomization process**	1.1 Was the allocation sequence random?	Y	
	1.2 Was the allocation sequence concealed until participants were enrolled and assigned to interventions?	NI	
	1.3 Did baseline differences between intervention groups suggest a problem with the randomization process?	Y	Baseline FM-UE: VR: 67.8, Control: 49.0
	**Risk of bias judgement**	**High**	
**Bias due to deviations from intended interventions**	2.1. Were participants aware of their assigned intervention during the trial?	NI	
	2.2. Were carers and people delivering the interventions aware of participants' assigned intervention during the trial?	NI	
	2.3. If Y/PY/NI to 2.1 or 2.2: Were there deviations from the intended intervention that arose because of the experimental context?	N	
	2.4 If Y/PY to 2.3: Were these deviations likely to have affected the outcome?	NA	
	2.5. If Y/PY/NI to 2.4: Were these deviations from intended intervention balanced between groups?	NA	
	2.6 Was an appropriate analysis used to estimate the effect of assignment to intervention?	NI	

	2.7 If N/PN/NI to 2.6: Was there potential for a substantial impact (on the result) of the failure to analyse participants in the group to which they were randomized?	NI	
	**Risk of bias judgement**	**High**	
**Bias due to missing outcome data**	3.1 Were data for this outcome available for all, or nearly all, participants randomized?	Y	
	3.2 If N/PN/NI to 3.1: Is there evidence that result was not biased by missing outcome data?	NA	
	3.3 If N/PN to 3.2: Could missingness in the outcome depend on its true value?	NA	
	3.4 If Y/PY/NI to 3.3: Is it likely that missingness in the outcome depended on its true value?	NA	
	**Risk of bias judgement**	**Low**	
**Bias in measurement of the outcome**	4.1 Was the method of measuring the outcome inappropriate?	N	
	4.2 Could measurement or ascertainment of the outcome have differed between intervention groups?	N	
	4.3 Were outcome assessors aware of the intervention received by study participants?	NI	
	4.4 If Y/PY/NI to 4.3: Could assessment of the outcome have been influenced by knowledge of intervention received?	N	
	4.5 If Y/PY/NI to 4.4: Is it likely that assessment of the outcome was influenced by knowledge of intervention received?	NA	
	**Risk of bias judgement**	**Low**	
**Bias in selection of the reported result**	5.1 Were the data that produced this result analysed in accordance with a pre-specified analysis plan that was finalized before unblinded outcome data were available for analysis?	N	
	5.2 ... multiple eligible outcome measurements (e.g., scales, definitions, time points) within the outcome domain?	N	
	5.3 ... multiple eligible analyses of the data?	N	
	**Risk of bias judgement**	**Some concerns**	
**Overall bias**	**Risk of bias judgement**	**High**	

**Unique ID**	Huang et al. (2023)	**Study ID**	Huang et al. (2023)	**Assessor**	
**Ref or Label**	Huang et al. (2023)	**Aim**	Assignment to intervention (the 'intention-to-treat' effect)		

Experimental		Comparator		Source	
**Outcome**		**Results**		**Weight**	1

Domain	Signalling question	Response	Comments
**Bias arising from the randomization process**	1.1 Was the allocation sequence random?	Y	Subject randomly assigned to the groups.
	1.2 Was the allocation sequence concealed until participants were enrolled and assigned to interventions?	Y	
	1.3 Did baseline differences between intervention groups suggest a problem with the randomization process?	N	Groups comparable at the baseline.
	**Risk of bias judgement**	**Low**	
**Bias due to deviations from intended interventions**	2.1. Were participants aware of their assigned intervention during the trial?	Y	Due to the context.
	2.2. Were carers and people delivering the interventions aware of participants' assigned intervention during the trial?	Y	
	2.3. If Y/PY/NI to 2.1 or 2.2: Were there deviations from the intended intervention that arose because of the experimental context?	N	
	2.4 If Y/PY to 2.3: Were these deviations likely to have affected the outcome?	NA	
	2.5. If Y/PY/NI to 2.4: Were these deviations from intended intervention balanced between groups?	NA	
	2.6 Was an appropriate analysis used to estimate the effect of assignment to intervention?	Y	ITT
	2.7 If N/PN/NI to 2.6: Was there potential for a substantial impact (on the result) of the failure to analyse participants in the group to which they were randomized?	NA	
	**Risk of bias judgement**	**Low**	
**Bias due to missing outcome data**	3.1 Were data for this outcome available for all, or nearly all, participants randomized?	N	10% dropout in each group at post-intevention assessment.
	3.2 If N/PN/NI to 3.1: Is there evidence that result was not biased by missing outcome data?	Y	
	3.3 If N/PN to 3.2: Could missingness in the outcome depend on its true value?	NA	
	3.4 If Y/PY/NI to 3.3: Is it likely that missingness in the outcome depended on its true value?	NA	
	**Risk of bias judgement**	**Low**	
	4.1 Was the method of measuring the outcome inappropriate?	N	

Bias in measurement of the outcome	4.2 Could measurement or ascertainment of the outcome have differed between intervention groups?	N	
	4.3 Were outcome assessors aware of the intervention received by study participants?	N	Blinded assessor.
	4.4 If Y/PY/NI to 4.3: Could assessment of the outcome have been influenced by knowledge of intervention received?	NA	
	4.5 If Y/PY/NI to 4.4: Is it likely that assessment of the outcome was influenced by knowledge of intervention received?	NA	
	**Risk of bias judgement**	**Low**	
**Bias in selection of the reported result**	5.1 Were the data that produced this result analysed in accordance with a pre-specified analysis plan that was finalized before unblinded outcome data were available for analysis?	Y	Study registered at ClinicalTrials.gov (NCT03086889)
	5.2 ... multiple eligible outcome measurements (e.g., scales, definitions, time points) within the outcome domain?	N	
	5.3 ... multiple eligible analyses of the data?	N	
	**Risk of bias judgement**	**Low**	The research protocol was registered with ClinicalTrials.gov ID: NCT03135418).
**Overall bias**	**Risk of bias judgement**	**Low**	Patients were randomly divided into two groups, VR or control, with stratified randomization according to age, sex and stroke onset, using an online randomization website. No significant differences were found between the groups for any outcome measures before the training. An online randomization website. Both patients and outcome assessors were masked, which was achieved by using sham VR therapy with the control group and the outcome assessor being blinded to the groups. During the study, 10 patients from the VR group and nine patients from the control group discontinued their sessions. The research protocol was registered with ClinicalTrials.gov ID: NCT03135418).
